# A multielectrode array microchannel platform reveals both transient and slow changes in axonal conduction velocity

**DOI:** 10.1038/s41598-017-09033-3

**Published:** 2017-08-17

**Authors:** Rouhollah Habibey, Shahrzad Latifi, Hossein Mousavi, Mattia Pesce, Elmira Arab-Tehrany, Axel Blau

**Affiliations:** 1 Department of Neuroscience and Brain Technologies (NBT), Fondazione Istituto Italiano di Tecnologia (IIT), Via Morego 30, 16163 Genoa, Italy; 2 Department of Neurology, David Geffen School of Medicine, University of California Los Angeles, Los Angeles, California, USA; 3 Department of Computer and Software Engineering, Polytechnique Montréal, Montréal, QC H3C 3A7 Canada; 4 Department of Nanophysics (NAPH), Fondazione Istituto Italiano di Tecnologia (IIT), Via Morego 30, 16163 Genoa, Italy; 5 Laboratoire d’Ingénierie des Biomolécules, Université de Lorraine, 2 Avenue de la Forêt de Haye, 54504 Vandoeuvre-lès-Nancy Cedex, France

## Abstract

Due to their small dimensions, electrophysiology on thin and intricate axonal branches in support of understanding their role in normal and diseased brain function poses experimental challenges. To reduce experimental complexity, we coupled microelectrode arrays (MEAs) to bi-level microchannel devices for the long-term *in vitro* tracking of axonal morphology and activity with high spatiotemporal resolution. Our model allowed the long-term multisite recording from pure axonal branches in a microscopy-compatible environment. Compartmentalizing the network structure into interconnected subpopulations simplified access to the locations of interest. Electrophysiological data over 95 days *in vitro* (DIV) showed an age-dependent increase of axonal conduction velocity, which was positively correlated with, but independent of evolving burst activity over time. Conduction velocity remained constant at chemically increased network activity levels. In contrast, low frequency (1 Hz, 180 repetitions) electrical stimulation of axons or network subpopulations evoked amplitude-dependent direct (5–35 ms peri-stimulus) and polysynaptic (35–1,000 ms peri-stimulus) activity with temporarily (<35 ms) elevated propagation velocities along the perisomatic branches. Furthermore, effective stimulation amplitudes were found to be significantly lower (>250 mV) in microchannels when compared with those reported for unconfined cultures (>800 mV). The experimental paradigm may lead to new insights into stimulation-induced axonal plasticity.

## Introduction

High-level brain functions emerge from the real-time interaction of interconnected neural networks. Although axonal branches are mainly considered as signal transmission lines between neurons, they can also directly participate in information processing. For instance, recent evidence suggests that axons are involved in neural computations by changing the propagation timing, by broadening the action potential (AP) waveform at the presynaptic terminal, thereby enhancing synaptic transmission, and by analogue coding through the transmission of subthreshold postsynaptic potentials^1–3^. To determine whether axonal function affects the overall network activity dynamics requires access to subcellular recordings from these lean projections with millisecond resolution^4^.

Standard microelectrode arrays (MEAs) or high-density complementary metal oxide semiconductor (CMOS)-based MEAs are commonly used for non-invasive multisite extracellular recordings from cultured neurons^5^ and their axons^6, 7^. However, capturing signals from thin axonal branches with significantly lower extracellular signal amplitudes is more challenging when compared to other cellular compartments like somata and dendrites^6^. Recent studies of axonal biophysics therefore aligned polydimethylsiloxane (PDMS) microchannel devices with electrodes of commercial or custom-made MEAs^8, 9^ or CMOS-based MEAs^10, 11^ to both guide axons and to create an electrically isolated and more stable cellular microenvironment. This strategy, which was previously exploited for the comprehensive analysis of axonal biology^12, 13^, increases the extracellular sealing resistance and thus amplifies extracellular potentials, thereby significantly improving the signal to noise (SNR) ratio^10, 14, 15^. Multisite recording or stimulation with paired devices allowed studying axonal signal properties and activity-dependent changes in axonal signal conduction velocities under normal and chemically or electrically stimulated conditions^10, 11, 16, 17^.

All aforementioned studies primarily focused on young axons at a few days *in vitro* (DIV) and reported that higher activity levels such as bursts or electrically evoked activity decrease the AP propagation velocities along the microchannel^11, 16^. In contrast, our long-term study revealed an increase in axonal AP propagation velocity in parallel to the overall activity increase with culture age. We compared the effect of individual burst features and of chemically or electrically evoked activity on axonal signal propagation features in acute and chronic experiments. The chosen technological approach of combining MEAs with transparent microchannel devices tailored for segregating axons from 8 interconnected sparse neural subpopulations allowed for this comprehensive morphology-electrophysiology analysis on low-density axonal populations over a period of 95 days. The described platform and results may provide the basis for gaining new insights into how axonal activity processes and controls neural output.

## Methods

Tissue extraction from animals was carried out in accordance with the guidelines established by the European Communities Council (Directive of November 24, 1986) and was approved by the National Council on Animal Care of the Italian Ministry of Health.

### PDMS device fabrication

The microchannel device fabrication procedure was discussed in detail previously^15^. Briefly, an SU-8 template defining device compartment geometries was fabricated in two layers on a 4″ silicon wafer (Si-Mat). SU-8 5 and SU-8 50 (MicroChem) were subsequently spin coated on the wafer to generate two patterned layers of different heights (5 µm for microchannels and 100 µm for reservoirs). SU-8 layer thickness was controlled by the spinning speed (Ws-650Sz Spin Coater, Laurell Technologies). The thin and thick SU-8 layers were photo-patterned in a mask aligner (MJB4, SUSS MicroTec) with separate chrome (Photronics Ltd) or printed high-definition transparency masks (Repro S.r.l.) to define elevated SU-8 stripes and reservoirs. Pre-, post- and hard-bake as well as SU-8 development protocols were followed as suggested in the product datasheets (MicroChem). Physical dimensions of the final structures were determined by a stylus profiler (Wyko NT1100, Veeco) and quantitative microscopy (Leica DM IL LED Inverted, Leica Microsystems CMS GmbH) through Zeiss Axiovision software (v 4.8) measurements. PDMS pre-polymer and curing agent (Sylgard 184, Dow Corning) were mixed (10:1), degassed and poured on the original SU-8 template or an epoxy copy thereof (Epox A cast 655, Smooth-On). A laser copier transparency was placed on top of the PDMS to level the device thickness by squeezing extra PDMS out of the cavities. A thin layer (~100 µm) of PDMS was left between the transparency and the template’s highest structures to provide closed somal reservoirs in place of open reservoirs. PDMS was cured at 80 °C for 20 min, peeled-off from the template, and four big seeding cavities were manually punched at the two opposite outer corners of each small somal reservoir. The final device thus featured four big round-shaped seeding cavities with openings from the top and two quasi-closed somal reservoirs, which were connected through 8 microchannels. The individual steps and device features and dimensions are found in Suppl. Fig. 1 and Video 1.

### Cell preparation and device loading

Where not stated otherwise, chemicals were bought from Gibco/Life Technologies/Thermo Fisher Scientific. Pregnant Sprague Dawley rats (CD IGS, Charles River) were anesthetized and sacrificed by cervical dislocation 18 days after conception. Following standard tissue dissociation protocols^18^, their embryos (E17/E18) were harvested, put on ice in Hank’s balanced salt solution (HBSS) and decapitated. After removing the meninges, the hippocampi were extracted, minced and transferred to fresh HBSS and dissociated into single cells using 0.25% (w/v) trypsin in HBSS buffer. After incubation for 10 minutes at 37 °C, the trypsin was deactivated by 0.25 mg/mL (final concentration) soybean trypsin inhibitor along with 0.01% (w/v) DNase (Sigma). Cell suspensions were prepared by sequential trituration (15–20 times) using three fire-polished Pasteur pipettes with decreasing diameters. Cells were then centrifuged at 200 g for 5 minutes and the pellets were resuspended in Neurobasal medium (NBM) containing 2% B-27 serum-free supplement, 1 mM penicillin/streptomycin and 2 mM Glutamax.

**Figure 1:**
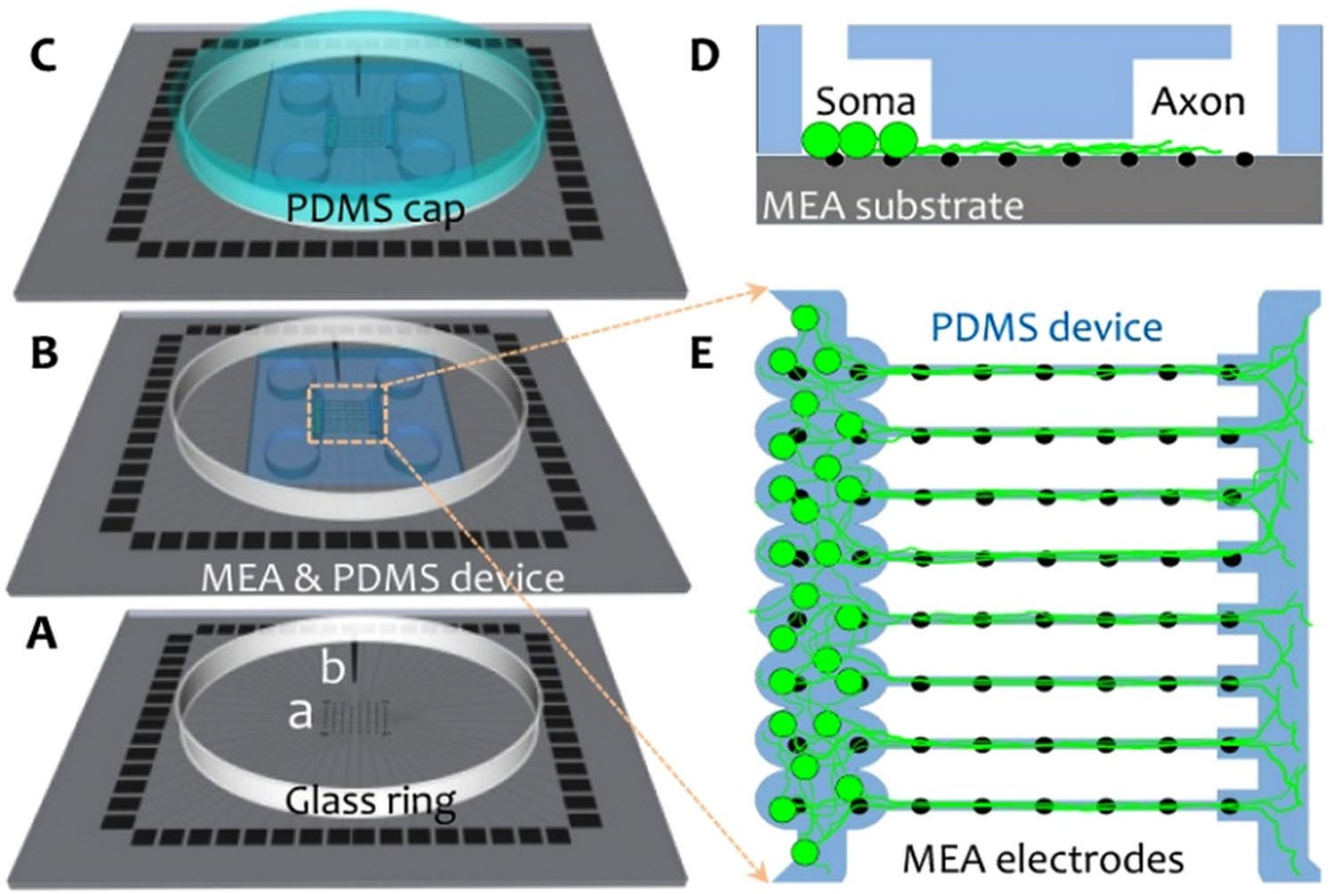
Coupling a PDMS microchannel device with a MEA. (**A**) MEA device with 59 recording electrodes (a) and one counter electrode (b). A glass ring (r  =  30 mm, height  =  5 mm) attached to the MEA substrate retains the cell culture medium. (**B**) The PDMS microchannels were aligned with the MEA electrodes and cells were seeded through one of the 4 device openings. (**C**) After seeding the cells and rinsing the whole PDMS device with cell culture medium, a custom-made PDMS cap sealed the culture to prevent evaporation and contamination. (**D** and **E**) cross-section and top view sketch of a network inside a coupled MEA-PDMS device. In each microchannel, five electrodes recorded from axons, and in each reservoir module, two electrodes recorded from the corresponding neuronal subpopulation.

With the exception of small variations, the general protocols for device alignment with the microelectrodes and cell seeding were discussed previously^15^. Briefly, PDMS devices were placed on a glass-slide and were baked in an oven at 110 °C for 2 hours to finalize the cross-linking of uncured PDMS oligomers. All devices were placed on a glass-slide and autoclaved (120 °C, 20 min). The device was manually aligned with the 60 MEA electrodes (30/200 ir, Multi Channel Systems) under a light microscope (5x) to match five MEA electrodes with each microchannel and two electrodes with each reservoir module. Then, the substrate surface (MEA, cover slip or Petri dish) was hydrophilized by oxygen plasma (3 min, 60 W, 2.45 GHz, 0.4 mbar O_2_; femto, Diener) and coated with 5 µl of a 0.1 mg/ml poly-D-lysine (PDL) and 0.05 mg/ml laminin mixture in MQ water. PDMS devices on cover slips and Petri dishes were used as controls for immunostaining and time-lapse imaging. After drying the coating in a vacuum chamber, the whole device was washed three times with sterile MQ water (each time 10 min). Air bubbles trapped in the microchannels were removed by filling the MEA ring with MQ water and vacuuming it for a few seconds. The MQ water in the microchannels was later replaced with cell culture medium by diffusion during over-night incubation (5% CO_2_, 37 °C, 95% RH). Rat cortical neurons (8,000 cells/μl) were added through one of the large seeding cavities. Cells were distributed homogenously in all reservoir modules with a maximum number of 200 cells per reservoir module (1,600 cell/device). Extra cells were removed from the seeding cavities and 1.5 ml pre-warmed serum-free cell culture medium was added to each MEA or coverslip in a 12-well plate. The MEA ring was then sealed with a custom-made gas-permeable PDMS cap (Fig. 1)^19^. Between experiments, the cultures were stored in a regular humidified incubator (5% CO_2_, 37 °C, 95% RH). The electrophysiology recording, microscopy, and culture medium-exchange days are given in Suppl. Table 1. To picture all network modules, 9 and 16 mosaic bright-field microscopy images were captured with 10x and 20x objectives, respectively, and then stitched together manually in ImageJ (Suppl. Fig. 2, Video 2 and 3).

### MEA electrophysiology

To record cortical network activity or electrically stimulate the cultures, we used a commercially available 60-channels MEA filter-amplifier (0.1 Hz–25 kHz, 1200 x amplification, MEA1060-upright-standard) with an A/D conversion card (64-channels, 25 kHz sampling frequency/channel, PCIbus) and a software user interface (MC_Rack) provided by Micro Channel Systems (MCS). MEA cultures were divided into three groups. The first group included 7 MEA cultures for long-term electrophysiology and bright-field microscopy (13 DIV to 95 DIV). The data from these cultures were used for the analysis of spike and burst frequencies, spike propagation velocities and activity-velocity correlations over time. The activity of each culture was acquired on different days (Suppl. Table 1). The second group of MEA cultures (n  =  8) was used for electrical stimulation between 14 DIV to 75 DIV. Activity was recorded before, during and after each stimulation trial. The third group of MEA cultures (n  =  4) served to study the effect of bicuculline on activity and propagation velocity. During all recordings, the temperature was kept constant (37 °C) by a built-in thermal sensor and heating element controlled by an external temperature controller (HC-1, MCS). Raw signals were recorded and analyzed offline. All signals were filtered by a second-order Bessel high-pass filter (cut-off at 200 Hz). The timestamps of the spikes in the filtered data streams were detected by a negative threshold (−4.5 StDev of the peak-to-peak noise). After transforming the spike trains to time stamps (NeuroExplorer, Nex Technologies), the mean spike frequency on each electrode was extracted as spikes/s. Timestamps were also used for extracting the propagation velocity between electrode pairs.

### Electrical and chemical stimulation

Voltage-driven biphasic stimuli (+then-,100 µs phase duration, 1 Hz) were applied to an electrode in a reservoir module for somal stimulation or to the first microchannel electrode for axonal stimulation by an 8-channel stimulus generator (MCS, STG4008) and control program (MCS, MC_Stimulus II). Three-minute baseline recordings preceded and followed each stimulation experiment not lasting more than 30 min in total. Three different stimulation amplitudes (250, 500 and 1000 mV) were tried on 12 microchannels and 12 reservoir modules of 8 different MEA cultures.

Four cortical cultures were stimulated with bicuculline. After a 15 min baseline recording, half of the cell culture medium was drained, mixed with bicuculline (60 μM), returned to the MEA and incubated for another 15 min in the incubator before performing another 15 min recording. Somal and axonal activity and activity propagation velocities along axons were compared between baseline and bicuculline-treated conditions.

### Immunofluorescence staining

Cell morphology was characterized by immunofluorescence on cultures prepared on 18 mm coverslips or 35 mm polystyrene Petri dishes (BD Falcon Tissue Culture Dishes, 353001). PDMS devices were removed before fixation by detaching one side of the device and then peeling it off by continuous bending to avoid damage to the axonal branches in the microchannels. The cell culture medium was then drained, and the culture was washed once with warmed 1% phosphate buffered saline (PBS). Cortical networks were fixed with 4% paraformaldehyde (PFA) and 4% sucrose solution (10 min), permeabilized with 0.2% Triton X-100 in 1% PBS (10 min), and incubated with blocking buffer (2% goat serum (GS) and 3% bovine serum albumin (BSA) in 1% PBS) for 30–45 min. After incubation with primary antibodies (1 h at RT) and washing them three times with 1% PBS, they were incubated with secondary antibodies (1 h at RT). Cultures were labeled for axons and somata/dendrites. The primary antibodies were a rabbit anti- MAP2 IgG (M3696, Sigma) and a mouse monoclonal antibody against the pan-axonal neurofilament (SMI-312R, BioLegend). The secondary antibodies were Alexa Fluor 488 (goat anti-mouse) and 633 (goat anti-rabbit), both from Molecular Probes. Finally, 10 µl of mounting solution (Thermo Fisher Scientific) including DAPI as a nuclear marker were added to each culture and covered by a coverslip. Fluorescence was observed using an inverted confocal microscope (Nikon A1+). Images were taken by an Optronics Microfire microscope camera (2-megapixel, MBF). To prepare large high-resolution images from the whole culture area, 20 mosaic images were taken (20x) and stitched and processed with ImageJ^20^.

### Statistical analysis

In each MEA culture, two electrodes recorded from one of the 8 reservoir modules, and five subsequent electrodes recorded activity propagation in the same axonal bundle in 200 µm intervals inside the microchannel. The mean number of spikes per second, the number of bursts per second, various burst features and timestamps of spikes recorded from individual electrode were extracted by NeuroExplorer (Nex Technologies) and analyzed statistically (IBM SPSS Statistics 22)^21^. Complex spike trains were considered as burst activity if they met the following criteria: 20 ms maximum inter-spike interval to start the burst, 10 ms maximum inter-spike interval to end the burst, 10 ms minimum inter-burst interval, 20 ms minimum burst-duration with at least 4 spikes in each burst^15^.

To compare activity changes over time in the reservoir or microchannels, we used a repeated measure analysis of variance (ANOVA) followed by a Bonferroni test. The activity on each individual recording electrode was compared at different recording DIVs. The spike frequency or burst features were averaged for the selected module (reservoir or microchannel) across all MEAs and were represented as spikes/s per electrode and bursts/s per electrode (mean  ±  standard error of the mean). Association between the activity in each reservoir module and its corresponding microchannel was evaluated by a Pearson r correlation analysis for all recording DIVs. This analysis was also used to study the relationship between normalized values of the activity (spikes/s) and the number of completely propagating spikes along each microchannel (Suppl. Table 4).

To calculate the propagation velocity along the five subsequent electrodes in each microchannel, the individual spike timestamps from each electrode were extracted and transferred to Matlab (MathWorks, R2014b). A Matlab script extracted propagating spikes, which travelled over the whole microchannel length and had a time delay of 0 ms to 2 ms between each 200 µm-pitched electrode pair (Suppl. M File 1). In accordance with this time delay, we only considered propagation speeds above 0.1 m/s. The average propagation velocity of each individual spike was derived from its velocities between each electrode pair at five subsequent electrodes. The mean propagation velocity in each microchannel was calculated by averaging the speed of all propagating spikes at each recording DIV (Suppl. Table 4). The changes in the mean velocities recorded from each microchannel over 95 DIV was compared by a repeated measure ANOVA followed by a Bonferroni test, and data is represented as mean  ±  StDev across all microchannels (n  =  42) on all MEAs (n  =  7) at each recording day. The velocity in the proximal and distal axons over the entire experiment was compared by a two-way ANOVA with repeated measures in one factor followed by a post hoc Tukey range test^21^. Because electrodes in the same microchannel usually recorded from different axons with different AP propagation velocities at the same recording day, we also performed a frequency domain analysis. The velocity between 0.1–1.2 m/s was divided by 0.1 m/s intervals into 11 domains. The number of propagating spikes in each interval was counted and averaged across all microchannels at each recording day (Suppl. Table 4). The frequency of each velocity domain was plotted for each DIV.

An activity-velocity correlation analysis was performed by a Pearson’s r test either for the whole data set (all DIVs; Suppl. Table 2) or for each day separately (Suppl. Table 3 **)**. Activity-velocity data from each module (reservoir or microchannel) at each DIV was represented as one data point. The same analysis was performed for the normalized activity and velocity data (Suppl. Table 4). Activity from each module at each DIV was normalized to the average activity recorded from the same module over the entire duration of the study. Normalization neutralizes the effect of large activity differences recorded from different cultures or microchannels. In bicuculline-treated cultures, the spike frequency, burst frequency and propagation velocity were compared with baseline values by a paired t test. Results were expressed as means  ±  StDev for reservoirs or microchannels of all MEAs.

The effect of electrical stimulation through an electrode in a reservoir module was studied by recording the activity from its electrode pair and the five subsequent electrodes in its adjacent microchannel. Similarly, the effect of stimulating axons through the electrode in a microchannel most proximal to the reservoir was studied by recording the activity from the four subsequent electrodes in the same microchannel. To compare the effect of the stimulation pulse amplitude on activity, the 1 s time-period between stimulation pulses was sub-divided into shorter time-windows. Spike timestamps of each time-window were extracted by a Matlab script and aligned with the timestamp of the corresponding stimulation pulse (Suppl. M File 2). For each time-window, the average activity was calculated across 12 experiments, each including 180 trials (3 min). The changes in the mean activity at baseline and for different time-windows after stimulation were compared by repeated measure ANOVA followed by a Bonferroni test. A between-groups comparison was performed by a two-way ANOVA followed by a post hoc Tukey range test. The effect of the stimulation pulse amplitude on reliably eliciting a response was studied within a 5–35 ms post-stimulus time window divided into 10 ms bins. The presence of at least one spike in each time-window was considered as a positive response and the lack of activity as a negative response. For each stimulus pulse amplitude, the percentage of positive responses was calculated for 180 trials and averaged across 12 different experiments. The effect of different stimulus amplitudes on spike frequency, fidelity of evoked response and propagation velocity was assessed by a two-way ANOVA followed by a post hoc Tukey range test. Propagating spikes on four electrodes distal to the stimulation electrode were extracted from different post-stimulus time-windows; the velocity of each electrode pair was averaged across 180 pulses and 12 experiments. The changes in the mean velocity at baseline and for different time windows after stimulation were compared by repeated measure ANOVA followed by a Bonferroni test. For all analyses, p  <  0.05 was considered to be significant.

## Results

**Figure 2:**
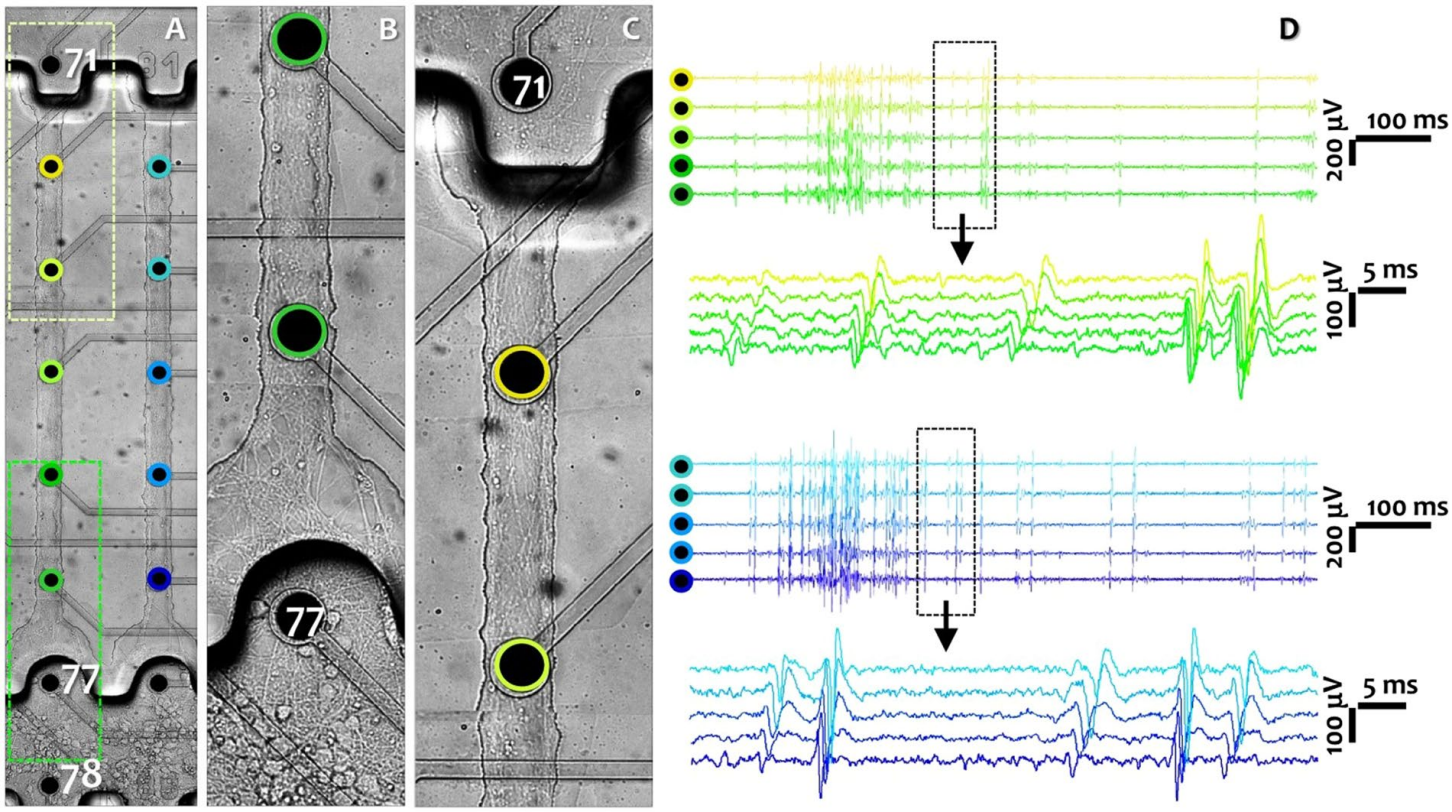
Imaging axonal morphology and recording its extracellular activity at 10 DIV. (**A**) Two reservoir modules (somal compartments, bottom) and microchannels with axonal projections. Axons are passing the neurite filtering region before reaching the microchannel entrance. Two electrodes are recording from each reservoir module. Five subsequent electrodes recorded from each microchannel. (**B)** Magnified view of one reservoir module and its dendrite buffer region (lower green rectangle in A). (**C)** Magnified view of counterpart compartment with exiting axons (upper yellow frame in A). (**D)** Long-term window of axonal activity (1 s) from subsequent electrodes of a wide (green circles in A and green signals in D) and narrow (blue circles in A and blue signals in D) microchannels. The dashed-rectangles (60 ms) zoom onto the propagating action potentials in a wide and a narrow microchannel (green and blue, respectively). Each panel shows different propagation velocities. The whole culture morphology at 10 DIV including all 8 modules is shown in Suppl. Fig. 2. Electrode pitch: 200 µm.

**Figure 3:**
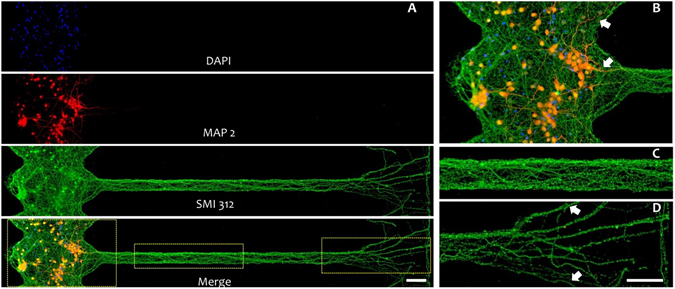
Immunofluorescence images from a cortical culture in a PDMS device on a glass coverslip at 10 DIV. (**A**) Somal reservoir (left), microchannel and counterpart reservoir (right); top to bottom: nuclei (DAPI, blue), somata and dendrites (MAP2, red), axons (SMI 312, green) and merged image. (**B**–**D**) Magnified view of merged image in A (yellow rectangles) showing a reservoir module with dendrites extending into the axonal diodes (white arrows), complex axonal bundle morphology inside the microchannel and branched-out axonal projections at the releasing point to the counterpart reservoir (white arrows). Scale bars: 30 µm. The overall culture with 8 reservoir modules and microchannels is shown in Suppl. Fig. 4.

200 µm thick PDMS devices featured three main compartments: reservoir modules, axonal diodes and narrow (25 µm) or wide (40 µm) microchannels of equal height (5 µm) and length (1 mm) (Fig. 2 and Suppl. Fig. 2). The somal compartment was composed of eight interconnected oval reservoir modules with a height of 100 µm. These modules guided cells to settle close to the diode-shaped microchannel entrance and partially separated neural subpopulations between channels (Figs 1E and 2A). 200 µm-wide semicircular openings with lower heights (5 µm) in front of the microchannel entrances were termed “axonal diodes”. Their purpose was two-fold. Firstly, they collected axonal bundles mainly from the corresponding reservoir module and guided them into the microchannels being aligned with the MEA electrodes (Fig. 2B). In addition, they acted as dendrite buffers to prevent shorter dendrites from entering into the microchannels, thereby ensuring a pure axonal population over the entire length of a microchannel (Fig. 3B and Suppl. Fig. 4). These axonal diodes thus facilitated the analysis of recorded data from the proximal parts of the microchannels and guaranteed axonal stimulation at the first electrode inside the microchannel. Likewise, channels ended in a 100 µm wide intermediate structure with the same height of the reservoir, which prevented axons from growing back into the microchannels after being released into the counterpart reservoir. These intermediate structures also prevented a sharp decrease in signal amplitude at the most distal electrode inside a microchannel (Figs 2C and 3D).

Due to device transparency, we could study both the axonal growth dynamics in the microchannels and neuronal network formation in the somal reservoir by light microscopy. At 3 DIV, cortical neurons started to expand their neurites into the axonal diodes. Within 7 to 10 days, axons grew through the entire microchannel and branched out into the counterpart reservoir (Fig. 3 and Suppl. Fig. 2). At later DIVs, axonal branches from more distant neurons entered into the microchannels and bundled into thick and complex axonal assemblies. These axonal assemblies tended to stay at the microchannel edges while individual branches crowded the central areas, especially those traversing the microchannel width (Fig. 3 and Suppl. Fig. 4).

**Figure 4:**
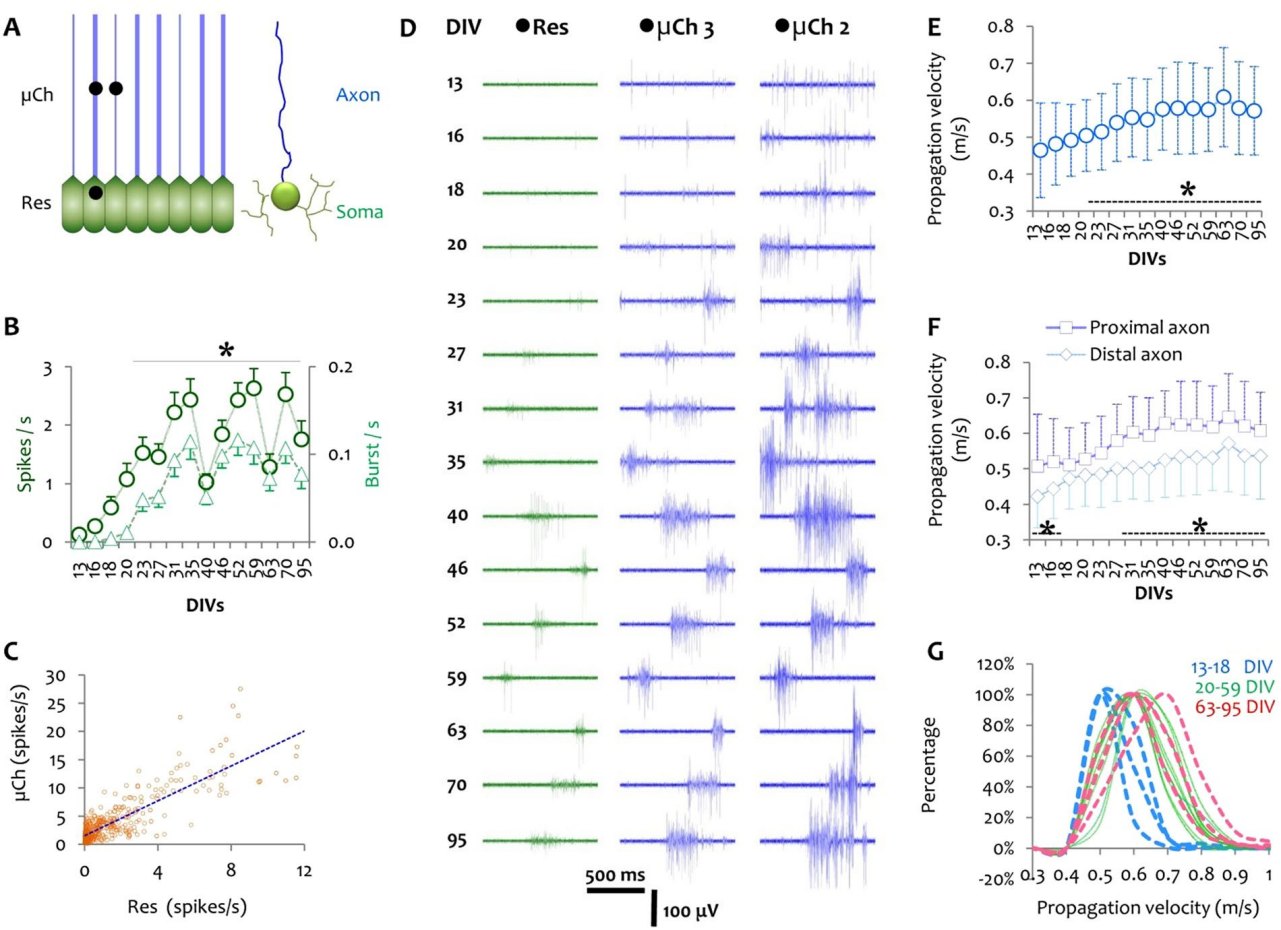
Long-term activity profile in different network modules and axonal propagation velocity over months. (**A**) Activity was recorded extracellularly by electrodes (dark disk) located in a reservoir module (green) or in narrow or wide microchannels (blue). (**B**) Average spike frequency (spikes/s) and burst frequency (bursts/s) in the reservoir modules (somata, green) between 13 DIV and 95 DIV. Data was pooled from reservoir modules (n  =  56; including 98 electrodes) of 7 cultures. The activity in each module at each recording DIV was averaged across all MEAs and was presented as mean  ±  S.E.M. (**C**) Correlation of the activity (spikes/s) between each reservoir module and its corresponding microchannel for 56 reservoir-microchannel modules at 15 different time points (n  =  840 data points). (**D**) Exemplary activity profile recorded from one reservoir electrode (green) and two (wide and narrow) microchannel electrodes (blue) over 95 DIV with their positions pointed out in A. (**E**) Long-term action potential propagation velocity along axons (n  =  42 microchannels) of 7 cultures. Mean propagation velocity for each microchannel calculated at each DIV by averaging the velocity of all propagating spikes in that microchannel. The total number of propagating spikes is plotted in Suppl. Fig. 6A. *p  <  0.05 *vs*. velocities at 13 DIV and 18 DIV. Velocity data is represented as mean  ±  StDev. **(F)** Average propagation velocity in proximal *vs*. distal segments of the same axonal bundles (n  =  42 microchannels). The first 500 μm of each microchannel were considered as the proximal section and the next 500 μm were considered as the distal segment. *p  <  0.05 *vs*. distal segment at each DIV. **(G)** Distribution of the velocity domains at each recording DIV. All velocities were categorized in 11 domains from 0.1 to 1.2 m/s (in 0.1 m/s intervals). The number of spikes belonging to each domain was counted in all microchannels at each DIV. The percentage of spikes within a specific velocity domain at each recording DIV was calculated (Suppl. Table 4). The three first and last recording DIVs are shown with blue and red dash lines, respectively.

Due to the small cross-sections of the microchannels, the amplifier was able to pick up the weak and thus otherwise difficult-to-capture extracellular potentials of axonal branches with signal-to-noise ratios between 2 and 35 (Fig. 2D). This allowed us to study the velocity of the propagating spikes by recording from subsequent points of the same axonal projections in one microchannel (Fig. 2D). Because the microenvironment was shielded from the bulk medium, the PDMS devices furthermore allowed us to keep small-world cortical networks functionally alive over months. First signals from axons inside of the microchannels appeared around 6 DIV as single spikes. Burst activity was observed around 10–13 DIVs in most microchannels (Fig. 2D and Suppl. Fig. 3). At older age, axonal activity was usually composed of single spikes and complex bursts with changes in burst duration and inter-burst intervals over months (Fig. 4 and Suppl. Fig. 2). Signals with different amplitudes (40 µV to 1,000 µV) and shapes (monophasic, biphasic, wide or narrow) were recorded in each microchannel (Fig. 2D). In particular, signals recorded from reservoir electrodes had significantly lower amplitudes (max  =  400 µV) than those recorded from microchannel electrodes (max  =  1,000 µV). This is shown in an exemplary recording at 10 DIV (Suppl. Fig. 3A) and in the activity evolution for one microchannel over time (Suppl. Fig. 2B). This amplification is related to the higher electrical impedance of the microchannel environment compared to the lower impedance of the reservoir modules with larger volume (Fig. 4D and Suppl. Fig. 2A)^17^.

### Network activity development in reservoirs and microchannels over time

The PDMS microchannel devices (Fig. 4A) allowed separating somal from axonal network activity over a period of 95 DIV (Fig. 4B and D). The average spike frequencies in the reservoirs (Res) increased sharply up to 23 DIV and, with the exception of a few fluctuations, remained almost constant at later DIVs (Fig. 4B). Signals recorded from adjacent microchannels started to synchronize from 23 DIV onward (Fig. 4D), concomitant with a significant increase in spike frequency and burst activity in the reservoir modules (p  <  0.01 *vs*. DIVs before; Fig. 4B and C). In general, spike and burst frequencies were higher on electrodes located in the microchannels compared to electrodes in the reservoir module (Fig. 4D). We furthermore found a strong correlation between the activity level in each reservoir module and its corresponding microchannel (Pearson’s r  =  0.84, p  <  0.01; Fig. 4C). Although signal amplitudes were higher in narrow microchannels compared to wide microchannels, microchannel width had no significant effect on spike frequencies (Suppl. Fig. 5A). At early DIVs, spike propagation along an axon could not always be sampled from all and, in particular, remote electrodes within a microchannel. However, the number of spikes successfully propagating over the entire length of the microchannels increased significantly over time (p  <  0.05; Suppl. Fig. 6A) and was moderately correlated with the overall activity level in the same microchannel (Pearson’s r  =  0.27, p  <  0.05; Suppl. Fig. 6B).

### Temporal development of the signal propagation velocity

The spike propagation velocity was calculated by dividing the constant electrode pitch (200 μm) by the delay of a signal to appear on two subsequent electrodes inside a microchannel. This allowed calculating the propagation velocity in different axonal segments (proximal or distal) at different DIVs. Propagation velocities in a microchannel could vary on the same DIV (StDev in Fig. 4E). More than 95% of the recorded velocities ranged between 0.3 – 1.2 m/s. A moderate shift of the peak in the velocity histogram was observed over time (from 0.5 m/s at 13–18 DIV to 0.6 m/s or 0.7 m/s at later DIVs; Fig. 4G). The average velocity increased over 95 DIV (0.46  ±  0.12 m/s at 13 DIV to 0.57  ±  11 at 95 DIV, p  <  0.05; Fig. 4E and G). Axonal conduction velocity reached its peak at 63 DIV (0.6  ±  0.13 m/s; Fig. 4E and G). In all microchannels, the average propagation velocity in the proximal section of an axon was higher than in its distal section, which was significant at DIVs later than 31 DIV (p  <  0.05, Fig. 4F). The microchannel width did not change the axonal spike propagation velocity, though (Suppl. Fig. 5B).

### Activity velocity correlation

**Figure 5:**
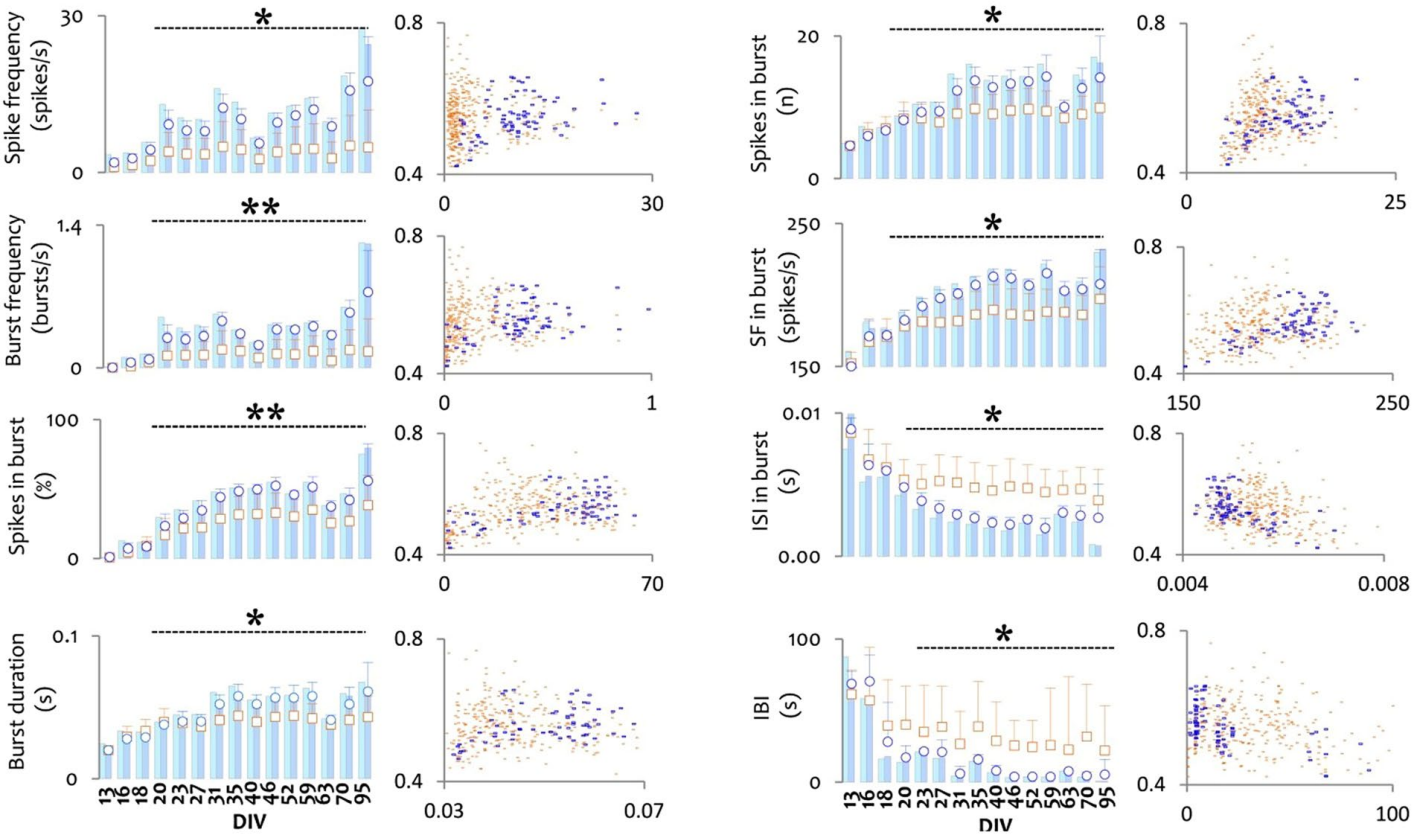
Axonal activity features over time and their correlation with the spike propagation velocity. Six different activity features were considered: spike frequency (spikes/s), burst frequency (bursts/s), average percentage of spikes in a burst (%), average burst duration (s), average number of spikes in a burst (n), average spike frequency in bursts (spikes/s), average inter-spike intervals in a burst (s), and average inter-burst intervals (s). Bar graphs: two blue bars (light blue and dark blue) at each recording DIV represent the average of a specific feature recorded from a narrow and a wide microchannel of one culture. For each DIV, blue circles represent the average of a variable across 6 microchannels of one culture (n  =  90 data points), orange circles the average over 7 cultures (n  =  42 microchannels and n  =  630 data points). Data are represented as mean  ±  StDev. Scatter plots: correlation analysis between each feature and the signal propagation velocity on data collected over three months from all cultures (brown dots: >400 data points) and one exemplary culture (blue dots). For each microchannel, the signal feature (x) and propagation velocity (y) was averaged over a 15 min recording window for each recording day to result in one data point (orange or blue dots). ISI: inter-spike intervals in a burst, IBI: inter-burst intervals. *p < 0.05 and **p < 0.01 *vs*. DIV 13–18 values (repeated measure ANOVA). P values and details on the correlation analysis are summarized in Suppl. Table 3.

To understand whether the overall network activity affects the propagation velocity, we studied the impact of the axonal activity level on the action potential propagation velocity along the same axon. The average activity in a microchannel (five electrodes) on each recording DIV and the average signal propagation velocity in a module were considered as one data point. A Pearson’s correlation analysis showed that the signal propagation velocity is independent of the general activity level (spike frequency) of the axonal branches (Pearson’s r  =  0.05, p  =  0.31; Fig. 5). However, we found a statistically significant correlation between each burst feature and the propagation velocity (Fig. 5). An increase in the mean burst frequency, the mean percentage of all spikes that compose a burst, the mean burst duration, the mean number of spikes in bursts and the mean spike frequency in bursts were all positively correlated with an increased propagation velocity (Suppl. Table 2 and Fig. 5). In contrast, an increase of the mean inter-spike intervals in bursts and of the mean inter-burst intervals was negatively correlated with the propagation velocity (Suppl. Table 2 and Fig. 5). Similar correlation features were observed between culture age and burst features. Except for a decrease in both the mean inter-spike interval per burst and the mean inter-burst interval, all other burst features increased with culture age (Fig. 5). These findings suggest that the correlation between burst features and propagation velocity can be indirectly related to culture age because the correlation analysis was performed on data covering the entire duration of the study (13 DIV to 95 DIV) as illustrated in Fig. 4E. To exclude changes in signal properties with culture age as a causal link to changes in the signal propagation velocity, we performed a similar analysis for axons of the same age, but with different spike frequency and burst frequency levels, which resulted in no correlation between changes in the signal features and the propagation velocity (Suppl. Table 3).

**Figure 6:**
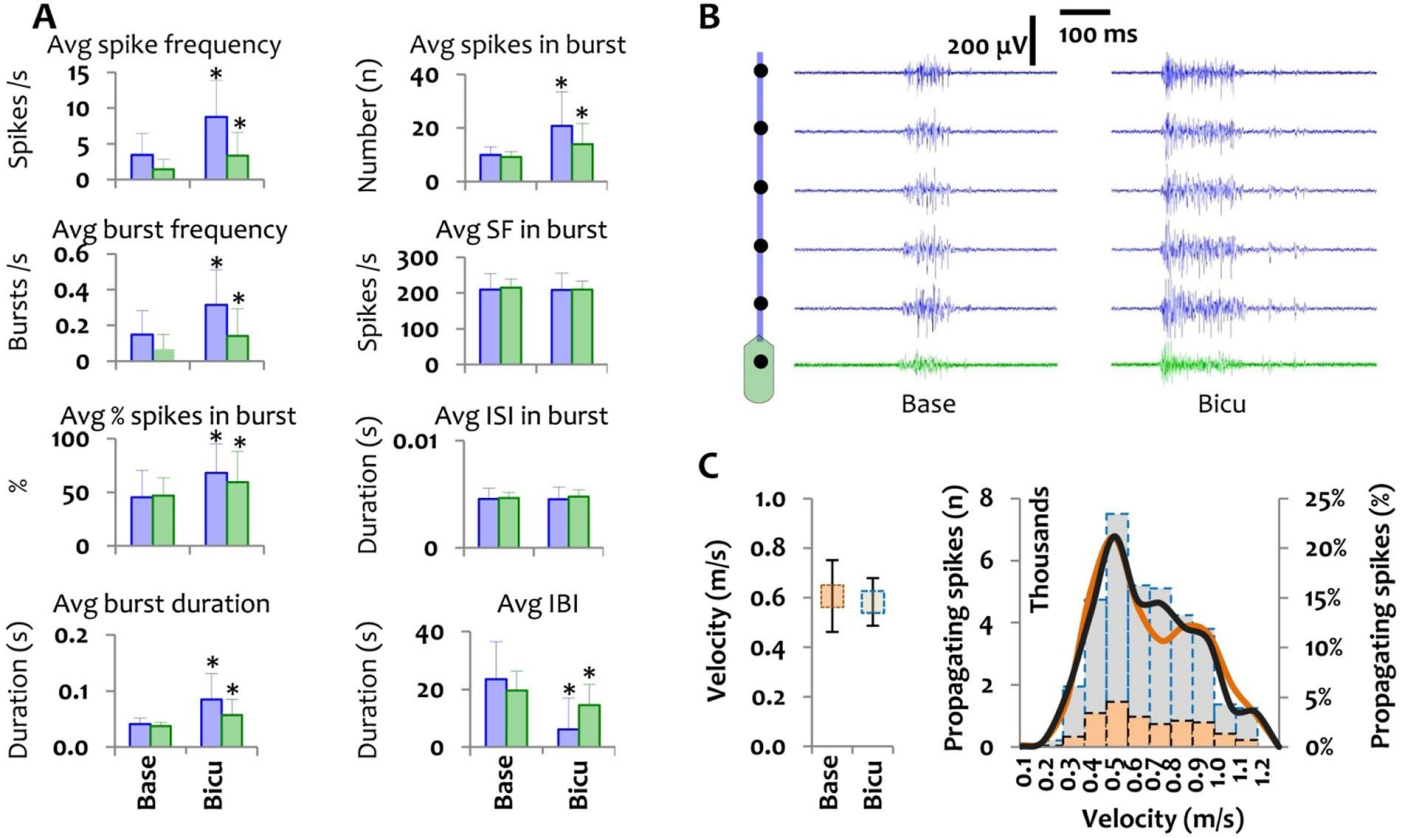
The effect of bicuculline on activity features and propagation velocity. (**A**) The effect of bicuculline on the overall activity in the soma (green bars) and axon (blue bars) expressed as mean spike frequency, mean burst frequency, mean percentage of spikes in a burst (%), mean burst duration, average number of spikes per burst, mean spike frequency (SF) in a burst, mean inter-spike interval (ISI) in a burst and mean inter-burst interval (IBI). Data are represented as the mean  ±  StDev of the baseline activity or after bicuculline treatment in four cortical cultures including 153 microchannel electrodes (blue bars and signals) and 56 reservoir electrodes (green bars and signals). (**B**) Exemplary recording profile of one network module including reservoir (green signals) and axons (blue signals) at baseline and after bicuculline treatment. (**C**) Propagation velocity in bicuculline-treated cortical cultures (n  =  4) at 24 and 25 DIVs. Left graph: average propagation velocity at baseline and after bicuculline treatment (n  =  32 microchannels). Right graph: effect of bicuculline treatment on the number (gray bars) and percentage (dark line) of propagating spikes in each velocity domain. The percentage of propagating spikes in each velocity domain at baseline (orange line) or after treatment with bicuculline (dark line) was calculated by dividing the number of propagating spikes in each velocity domain by the total number of propagating spikes in all velocity domains. Avg: average, Base: baseline, Bicu: bicuculline.

To test whether changes in the burst features can affect the signal propagation velocity along axons of the same age, we chemically increased network activity levels (mean spike and burst frequencies) by blocking the inhibitory GABA synapses with bicuculline (60 μM) (p < 0.01 *vs*. baseline; Fig. 6A). Other burst features such as mean percentage of spikes in bursts, mean burst duration and mean number of spikes per burst increased as well (p < 0.01 *vs*. baseline; Fig. 6A and B), whereas the mean inter-burst intervals decreased (p < 0.01 *vs*. baseline; Fig. 6A). The mean spike frequency in bursts and the mean inter-spike intervals in bursts did not change, though (Fig. 6A). This could be related to the concomitant increase in the mean number of spikes in a burst and the mean burst duration after bicuculline treatment. Likewise, the average propagation velocity was not affected by bicuculline (0.58  ±  0.09 m/s *vs*. 0.60  ±  0.14 m/s at baseline; Fig. 6C). Although bicuculline increased the number of propagating spikes in all velocity domains, the percentage of propagating spikes did not change when compared with baseline values (Fig. 6C).

### Electrical stimulation of confined axons and somata

**Figure 7:**
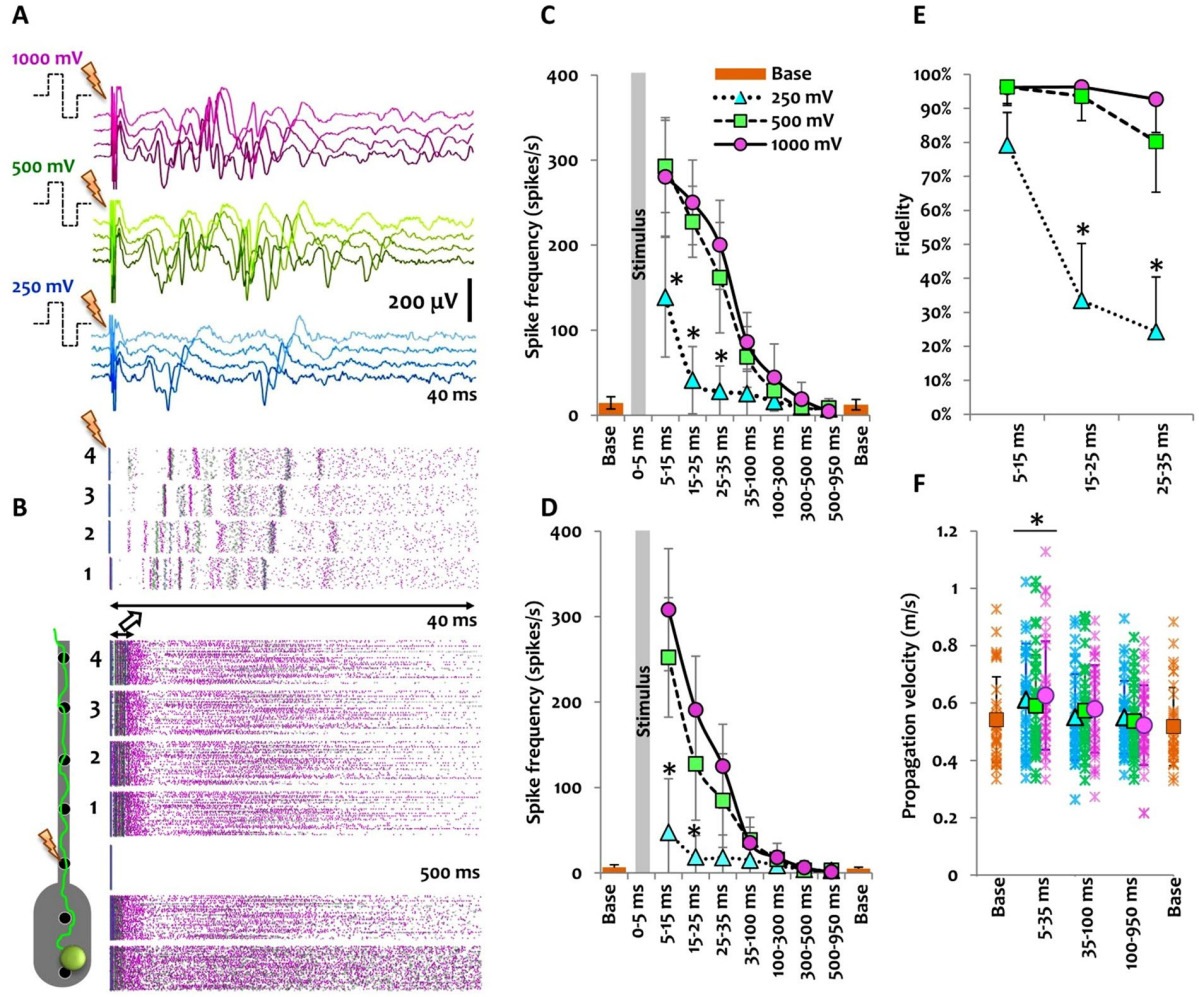
Local axonal stimulation in a microchannel and its effect on the signal propagation velocity. (**A**) From top to bottom: 40 ms post-stimulus responses to a symmetric biphasic (1 Hz;  ±  200 µs: first positive (+) phase (100 µs duration) followed by negative (−) phase (100 µs duration)) stimulus with 1,000 mV (red), 500 mV (green) or 250 mV (blue) amplitude. In all cases, the stimulation pulse was applied through the proximal electrode and the direct effect or evoked responses were recorded by four subsequent electrodes from the same axonal branch as shown in B. Darker colors indicate closer proximity of the recording electrode to the stimulation site. **(B)** Overlaid peri-stimulus raster plots for 100 biphasic stimulation pulses of 1,000 mV (red), 500 mV (green) or 250 mV (blue) amplitude. Lower panel: 500 ms peri-stimulus raster plot for four subsequent microchannel electrodes and two corresponding reservoir electrodes. The upper panel illustrates the first 40 ms of the same raster plot on four subsequent microchannel electrodes with reliable and densely packed responses over the first 15 ms, which also matches the exemplary recording profiles in A. In each raster plot, the responses to the 100 stimuli were arranged in 100 rows from top to bottom. (**C)** Axonal activity response to stimuli with different amplitudes during a 1 s peri-stimulus period was defined as the mean number of spikes per second for specific time-widows. The mean activity in each time-window was averaged across 12 experiments (for 4 cultures and 12 microchannels). In each experiment, three different stimulation amplitudes (180 trials each; 1 Hz) were applied to the same electrode. Baseline activity recorded from the same set of electrodes before and after each stimulation experiment. Data are represented as mean  ±  StDev. *p < 0.05 *vs*. baseline activity (Base) in the same microchannel. (**D)** Activity recorded from the corresponding reservoir module (one electrode pair in each reservoir) for the same experiments as in C. *p < 0.05 *vs*. baseline activity in the reservoir module. **(E)** The response reliability to certain stimulation pulse amplitudes was counted in three peri-stimulus time windows over 180 trials and the percentage of successful trials (at least one spike per time–window) was calculated. Individual points represent the average reliability percentage across 12 stimulation experiments for each stimulation pulse amplitude. *p < 0.05 *vs*. the response fidelity to 1,000 mV stimuli in the same time-window. (**F)** Propagation velocity in electrically stimulated axons for different pulse amplitudes (pink: 1,000 mV, blue: 500 mV, green: 250 mV, orange: baseline). Propagating spikes in different peri-stimulus time-windows were extracted and the average velocity was calculated across 12 experiments (mean  ±  StDev). In each time window, 36 points represent the average signal velocity at three axonal segments between electrodes 1 to 4 in B. *p < 0.05 *vs*. velocity at baseline.

Bicuculline stimulation increases the overall network activity with long-lasting effects. To better understand whether local and temporally limited stimuli change the axonal signal propagation velocity, we applied voltage pulses of different amplitudes either through an electrode in the reservoir (somata) or through the first electrode inside the microchannel (directly to axons). The minimum stimulation amplitude required to initiate a response in non-confined control cultures was 800 mV (data not shown). In contrast, the minimum pulse amplitude required to elicit a response in the reservoir or the microchannels was lower (500 mV and 250 mV, respectively; Fig. 7 and Suppl. Fig. 7).

To stimulate axons, we applied three different stimulation pulses (250, 500 and 1,000 mV;  ±  100 µs; 1 Hz) to the first electrode and studied the properties of the anterograde propagating signals recorded by four subsequent electrodes in 5 ms to 950 ms time windows after stimulation (Fig. 7). Microchannels tended to equally amplify both extracellularly recorded signals and stimulation artifacts. However, no differences in the duration of the stimulation artifact with or without microchannels could be observed. Although previous studies report on spike extraction as early as 2 ms post-stimulus by means of local curve fitting algorithms^22, 23^, we considered digitally high-pass-filtered (>200 Hz) activity from 5 ms onward to exclude any ambiguities. A direct response to all stimulation pulse amplitudes was recorded within a 5–15 ms post-stimulus window on four subsequent electrodes inside the microchannel and two electrodes in the corresponding reservoir module (Fig. 7A and B). The spike frequency was higher in response to 500 mV and 1,000 mV compared to 250 mV pulse amplitudes (p < 0.05; Fig. 7C and D) as can also be seen in the peri-stimulus raster plot at 5–15 ms (Fig. 7B). A sharp decrease in the activity response to a 250 mV stimulation pulse was already observed after 15 ms (p < 0.05 *vs*. 5–15 ms), while a significant decrease in activity response was observed only after 35 ms in response to 500 mV and 1,000 mV pulses (p < 0.05 vs. 5–15 ms; Fig. 7C and D). The slow response decay to higher pulse amplitudes can be attributed to complex polysynaptic signal transmission, which was not observed for 250 mV pulse amplitudes (Fig. 7B). The reliability of different pulse amplitudes to initiate an axonal response was evaluated by calculating the percentage of positive trials with at least one response in each 10 ms peri-stimulus time-window. The non-significant difference in the stimulus response fidelity after 5–15 ms was followed by a significant reliability decrease in response to a 250 mV stimulus after 15–35 ms (p < 0.05 *vs*. 1,000 mV in the same time-window; Fig. 7E). For 500 mV and 1,000 mV stimuli, the response reliability remained almost stable for 35 ms (Fig. 7E). To study the effect of electrically evoked local activity on the signal propagation velocity, we separated stimulation-induced activity (5–35 ms peri-stimulus) from the rest of the activity recorded between 35 ms and 950 ms in peri-stimulus or baseline recordings (Fig. 7F). All stimulation pulse amplitudes induced a moderate, yet significant increase in the signal propagation velocity at 5–35 ms peri-stimulus compared to the baseline velocity before or after a stimulation experiment (p < 0.05; Fig. 7F). The propagation velocity returned to baseline values at 35–950 ms peri-stimulus for all pulse amplitudes.

We also studied the effect of local electrical stimulation with different stimuli amplitudes for the reservoir module (Suppl. Fig. 7). One electrode in each reservoir module was used for electrical stimulation and the other for recording. The propagation of the evoked response was studied in the corresponding microchannel (Suppl. Fig. 7B). A direct response to 500 mV and 1,000 mV stimulation pulses could be recorded within a 5–15 ms post-stimulus window from both the reservoir electrode and the five subsequent microchannel electrodes (Suppl. Fig. 7A and B). In contrast, 250 mV stimulation pulses could not induce a direct response in more than 60% of the trials (p < 0.05 *vs*. 500 mV and 1,000 mV pulses; Suppl. Fig. 7E) and did not lead to any activity increase neither in the reservoir module nor in the microchannel within a 1 s post-stimulus time-window (p < 0.05 *vs*. 500 mV and 1,000 mV pulses; Suppl. Fig. 7C and D). A sharp activity increase during the 5–15 ms peri-stimulus window was observed after 500 mV and 1,000 mV pulses with a slow decay and return to baseline values within the 100–300 ms post-stimulus window (Suppl. Fig. 7C and D). Higher stimulation response reliability was observed on electrodes of the same reservoir 5–15 ms post-stimulus for 500 mV and 1,000 mV pulses with a slight and non-significant decrease in reliability after 15–35 ms in case of 500 ms pulses (Suppl. Fig. 7E). In the first 5–35 ms peri-stimulus, the evoked responses propagated with higher velocities along the microchannel (p < 0.05 *vs*. baseline before and p < 0.05 *vs*. baseline after stimulation; Suppl. Fig. 7F), whereas the propagation velocity during the subsequent 35–950 ms peri-stimulus window decreased to baseline values.

## Discussion and Conclusions

Advanced auxiliary technologies have proven their worth in better understanding how the nervous system processes information at cellular and network level. In this work, we exploited and optimized readily accessible technologies for isolating, imaging, recording from and stimulating axons to better understand their role in processing neural information. The concept of studying axonal biology in an isolated microchannel environment was introduced by Taylor *et al*.^13^ and later exploited in other contexts in different configurations^24^. When coupled with MEAs, such PDMS microchannel devices allowed to capture and amplify weak extracellular activity from axons, to measure the AP propagation velocity along axons, and to determine interactions between the overall network activity and that of APs^7, 8, 10^. By decreasing the device thickness to 200 µm, thereby enhancing its optical transparency, and by adding new morphology filter features to previous designs allowed us to perform the presented long-term electrophysiology and morphology studies from pure axonal branches. In all previous devices, the first 200–400 µm of the microchannels were occupied by a mixture of dendrites and axons, which interfered with axonal electrophysiology or molecular studies. In contrast, a diode-like neurite filtering gap between the microchannel entrance and the reservoir limited dendrite growth into the proximal microchannels. They furthermore collected the majority of axonal branches and guided them into the microchannels. This is particularly advantageous for low-density networks, where just a few axons find their way into a microchannel.

We furthermore compartmentalized the reservoir into eight modules to collect axonal branches from specific subpopulations of neurons in the reservoir. This allowed us to spatially locate and analyze the signal source. The strong correlation between the mean spike frequencies in each reservoir module and in its corresponding microchannel confirmed the ability of selectively and predictably guiding axons into the preferred microchannel. Minimizing the reservoir module dimensions helped in simplifying the network by decreasing the total required number of cells and by confining them to the recording areas. In contrast, previous designs with larger reservoir dimensions excluded the majority of neurons and network activity from analysis^11, 16, 17^. Finally, sealing the reservoir modules from top by a thin PDMS layer improved the signal to noise ratio and created a stable cellular microenvironment. All these features allowed us to study low density cortical networks with rich functional properties for more than three months.

The long-term electrophysiology data revealed an increase in the firing rate (spike frequency) both in somal and axonal modules. Equally, as already confirmed in previous studies on rat cortical neurons^25, 26^, burst activity increased during the first three weeks and stabilized thereafter. The activity increase with culture age was paralleled by a steady increase in the AP conduction velocity along the microchannel-confined axons. Interestingly, the conduction velocity in proximal axonal sections was found to be higher than in distal axonal sections over the entire duration of the study (Fig. 4). Although we did not find any correlation between the mean firing rate and the signal propagation velocity, burst features including burst frequency, mean percentage of spikes in a burst, mean burst duration, average number of spikes per burst, mean spike frequency (SF) in a burst, mean inter-spike intervals (ISI) in a burst and mean inter-burst intervals (IBI) were tightly correlated with the conduction velocity (Figs 4E and 5). This is in contradiction with recent work by Shimba *et al*., which suggested an activity-dependent decrease of the conduction velocity along microchannel-confined axons^16^. However, that study was performed on cortical cultures from mice instead of rats at a rather early developmental stage (10 DIV). Besides the difference in culture age and donor species, burst features in rat cortical neurons usually mature only after 14 DIV^25, 27^ and tend to vary thereafter age- and density-dependent^25, 26^.

To better understand why the conduction velocity correlates with the burst rate, we performed a correlation analysis between burst activity features and the signal propagation velocity at each day separately (Suppl. Table 3). No correlation between the levels of spontaneous activity and the conduction velocity was found. Secondly, we artificially increased the overall activity level by blocking the GABAergic synapses to find out whether disinhibited bursting activity increases the signal propagation velocity along axonal branches. Chemically induced burst activity by bicuculline in both the somal and axonal compartments did not change the conduction velocity along the axons either (Fig. 6). As previous discussions^1–3, 25, 26, 28, 29^ suggested, altered AP velocities might result from slow changes in the axonal membrane composition rather than from increased activity levels alone^1–3, 30, 31^. Our results confirm a slow, in this case age-related increase of the average AP propagation velocity along individual cortical axons, whereas elevated network activity by itself did not have any significant effect.

Because the inhibition of the GABAergic synapses increases activity in a difficult-to-control fashion over longer periods, it is difficult to study the effect of quick and defined changes in network activity on the axonal conduction velocity. We therefore resorted to a local stimulation strategy to test whether a particular stimulus feature induces a change in the axonal signal propagation velocity. We applied voltage pulses through individual MEA electrodes to either the axonal branches in the microchannels or to the mixture of somata and axons in the reservoir module and studied their effect on AP propagation velocity with millisecond temporal resolution along the axons (Fig. 7). The physical properties of the microchannels not only amplified the extracellularly recordable signal amplitudes, but also lowered the effective stimulation amplitudes to locally elicit action potentials in axons. Brief (100 µs) biphasic low-amplitude (±250 mV) voltage pulses had equal response fidelity in individual axons when compared with high-amplitude voltage pulses (≥800 mV) that are usually required in non-confined culture environments^23^. Such high-amplitude voltage pulses affected larger network areas instead (Fig. 7E).

Voltage pulses can either induce highly reproducible direct responses in a neuron in direct vicinity of the stimulation electrode with temporal onset delays below 25 ms or lead to more complex post-synaptic responses with longer delays and durations of up to 1,000 ms^22^. We measured the evoked response rate in different peri-stimulus time windows and observed a 20 to 30-fold activity increase during the first 35 ms that was followed by a steady decay to baseline activity levels during a 300 ms peri-stimulus period. Independent of the applied pulse amplitude, directly evoked responses propagated at higher speeds along axons. However, at 35–100 ms peri-stimulus, the signal propagation velocity returned to its baseline velocity, even though the activity was still higher than the baseline activity due to the involvement of complex polysynaptic responses (Fig. 7).

Taken together, these results suggest that the conduction velocity gradually increases with age and can be transiently increased in individual neurons through their direct low-frequency electrical stimulation, but is independent of overall network activity and unaltered in post-synaptic neurons. Similar results on local variability of conduction velocities in thin distal axons growing on CMOS-based high-density MEAs were reported by Bakkum *et al*.^6^. In that study, the antidromic signal propagation towards the soma was traced after applying biphasic voltage stimuli on distal axons. Even though age-dependent evoked signal velocity changes along the axon were observed, it was not possible to compare them with spontaneous network-intrinsic activity levels^6^. Recently, Lewandowska *et al*. coupled a CMOS-based high-density MEA with a microchannel device to isolate individual axons and to apply moderate to high frequency (10–160 Hz) voltage pulses to electrodes close to the somata. After increasing the stimulation frequencies beyond 50 Hz, both the axonal signal amplitude and conduction velocity decreased^11^. This effect was more apparent if the number of stimulations exceeded 500 repetitions. Conversely, in our model, 180 low-frequency voltage pulses (1 Hz), either applied to axonal bundles (Fig. 7) or to network subpopulations in the reservoir modules (Suppl. Fig. 7) increased the action potential propagation velocity in a 5–35 ms peri-stimulation window. Combined with previous studies^11^, our results therefore suggest that axons are able to change their conduction velocity as a function of the applied stimulation frequency. Because we did not observe any similar effect for the evoked polysynaptic responses, it seems that only directly evoked responses are prone to variations in stimulus frequencies^11^.

Direct electrical stimulation in different frequency regimes can lead to stimulation-induced plasticity in cortical and hippocampal networks, both *in vitro* and *in vivo* ^32–34^. Although we did not focus on stimulation-induced plasticity due to the absence of a post-synaptic network in the counterpart reservoir modules, previous studies have shown that low frequency stimulation (<1 Hz) of cultured cortical neurons induces changes in the spontaneous activity and functional connectivity of a network^30, 31, 35^. Even though plasticity is usually associated with synapses, stimulation-induced changes in the axonal conduction velocity may play a role in this context as well. Functional neural recovery by direct electrical stimulation already finds broad applications in clinical practice. For instance, it increases the activity of affected neural populations and induces cortical plasticity after stroke^36, 37^. Conversely, it has also been demonstrated to decrease activity levels in epilepsy^38^. Taking together previous evidence and our results, axonal compartments might be considered as a relevant target for stimulation-induced rehabilitation in the clinic.

## Electronic supplementary material

Supplementary Information
