# Supplementary material for: A multielectrode array microchannel platform reveals both transient and slow changes in axonal conduction velocity
Source: Sci Rep. 2017 Aug 17;7:8558. doi: 10.1038/s41598-017-09033-3 (PMC5561146; doi:10.1038/s41598-017-09033-3)
Supplement: Supplementary file 1 — Supplementary Information [file 41598_2017_9033_MOESM1_ESM.pdf]

# Supplementary Information

## A multielectrode array microchannel platform reveals both transient and slow changes in axonal conduction velocity

**Rouhollah Habibey[1], Shahrzad Latifi[2], Hossein Mousavi[3],**

**Mattia Pesce[1,4], Elmira Arab-Tehrany[5] and Axel Blau[1*]**

[1] Dept. of Neuroscience and Brain Technologies (NBT), Fondazione Istituto Italiano di Tecnologia (IIT), Via Morego 30, 16163, Genoa, Italy

[2] Dept. of Neurology, David Geffen School of Medicine, University of California Los Angeles, Los Angeles, California, USA

[3] Dept. of Computer and Software Engineering, Polytechnique Montréal, Montréal, QC H3C 3A7, Canada

[1,4] Dept. of Nanophysics (NAPH), Fondazione Istituto Italiano di Tecnologia (IIT), Via Morego 30, 16163, Genoa, Italy

[5] Laboratoire d'Ingénierie des Biomolécules, Université de Lorraine, 2 Avenue de la Forêt de Haye, 54504 Vandoeuvre-lès-Nancy Cedex, France

* Corresponding author's email address: axel.blau@iit.it

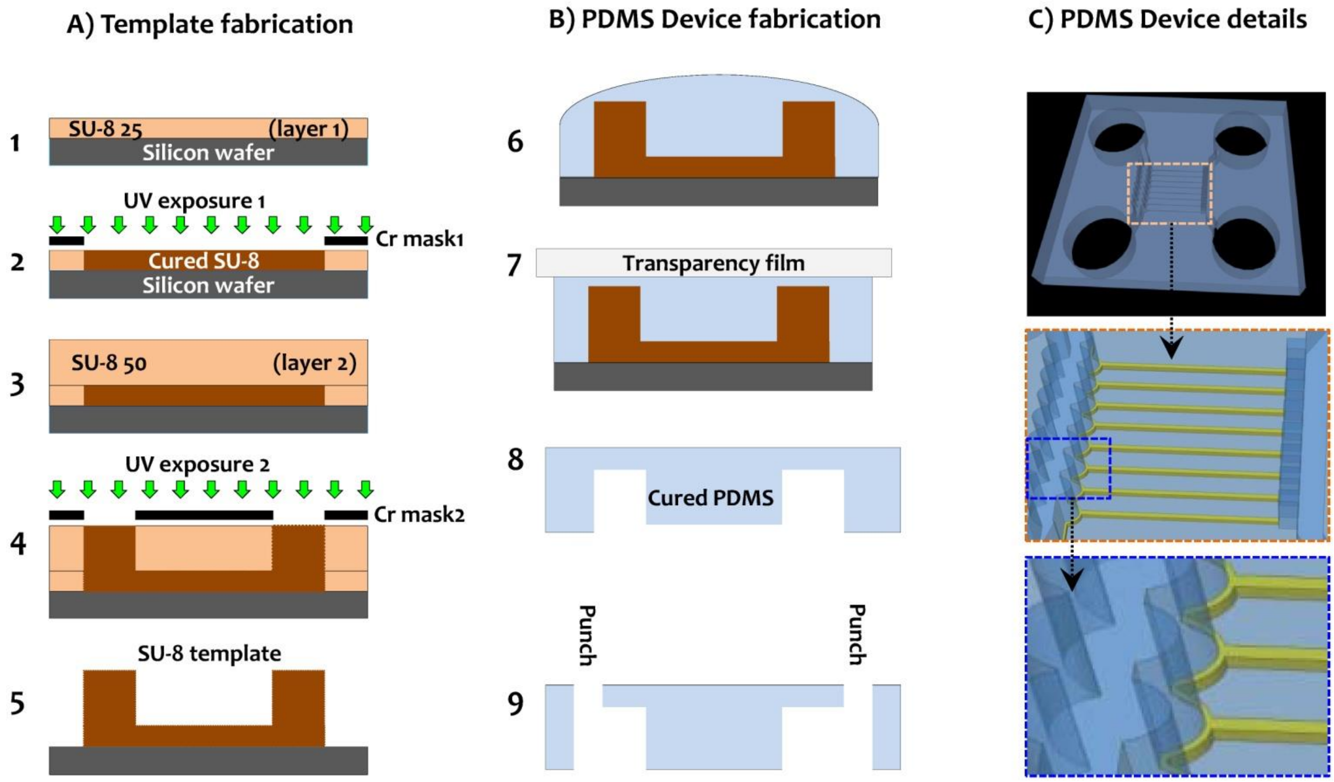


**Suppl. Fig. 1** SU-8 template and PDMS device fabrication steps. A) A SU-8 template was fabricated photolithographically in five steps: (1) spin coating of SU-8 25 on a silicon wafer with a thickness of <5 µm, (2) UV exposure of the SU-8 through the first chromium mask featuring all structural patterns, (3) spin coating SU-8 50 on top of the first layer with a thickness of 100 µm, (4) UV exposure of the second layer through the second chromium mask defining reservoirs and big pools only, and (5) developing the bi-layer SU-8 structures including the small strips for microchannels and taller areas for reservoirs. B) The PDMS microdevice was molded in four steps: (6) the PDMS pre-polymer with curing agent was poured on the surface of the template, (7) leveled by a transparency film to leave a thin PDMS layer on top of the elevated SU-8 structures, (8) cured and peeled off from the template, to finally (9) punch big pools at two corners of each reservoir. C) 3D sketch of the final PDMS device. The upper panel shows the whole device including four big cell seeding pools (r=1 mm), two opposite eight-module reservoirs (h=100 µm, l=300 µm and w=200 µm) and eight microchannels (h=5 µm, l=1,000 µm, and w=20-40 µm). The middle panel gives a magnified view onto the somal reservoir with 8 modules and corresponding microchannels. The lower panel shows three reservoir modules, neurite filtering cavities and microchannels. Structures with heights < 5 µm are depicted in yellow. Video 1 is presenting a 3D animation of the PDMS device coupled with a MEA.

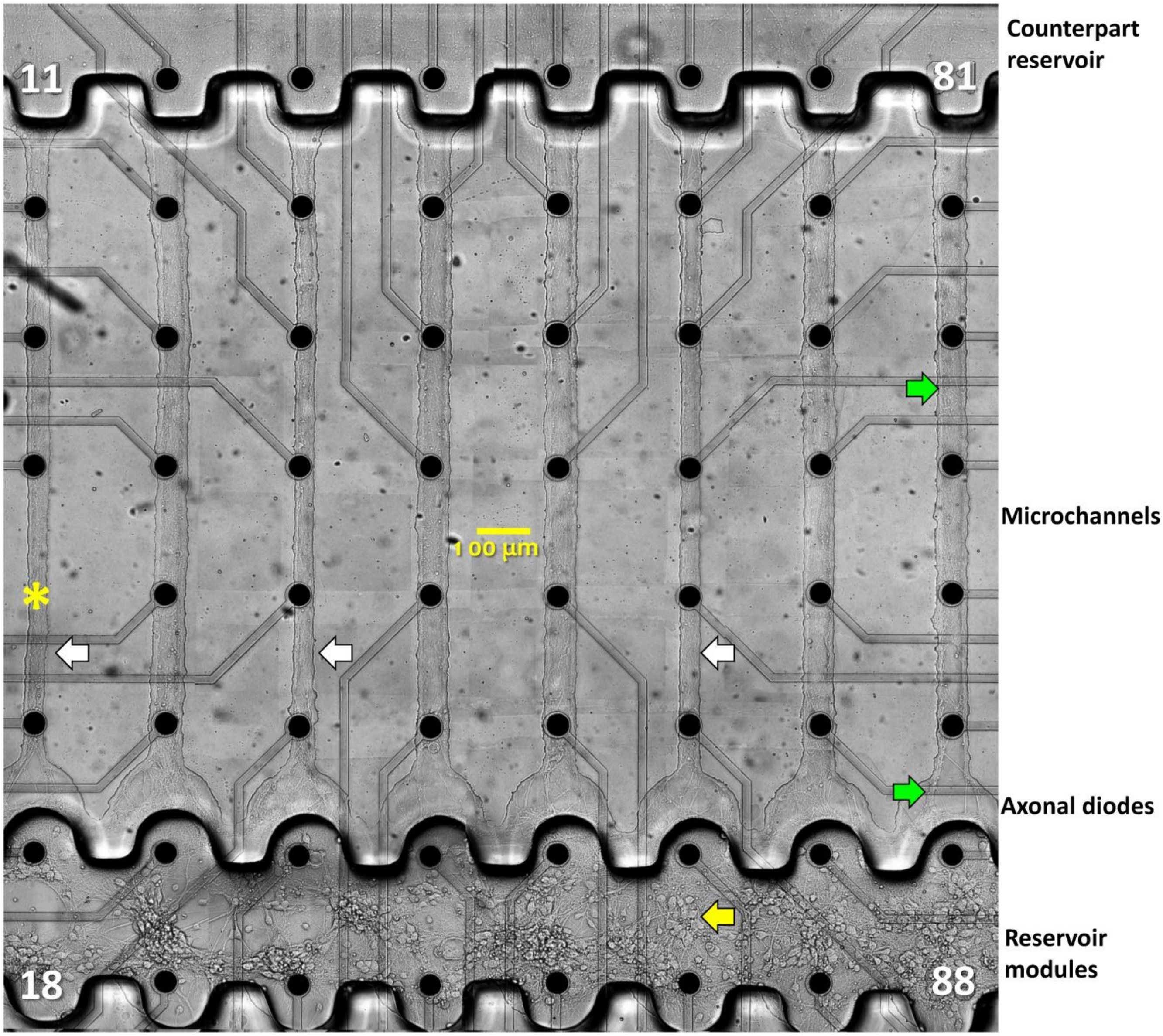


**Suppl. Fig. 2** High-resolution phase contrast microscopy of the entire network at 10 DIV by mosaic imaging and manual or automated stitching. To obtain high-resolution images that include details of axonal morphology inside the microchannels, 16 mosaic images were captured from different parts of the culture (20x) and then stitched together manually in ImageJ. Each PDMS device featured three narrow microchannels (25 µm width; white arrows) and five wide microchannels (~40 µm width). The yellow asterisk indicates the location of the missing electrode (electrode number 15), which is used as an off-center counter electrode (also see Video 1). Somata in the reservoir modules are exemplarily pointed out by a yellow arrow. Axonal projections in the axonal diodes and microchannels are shown by green arrows.

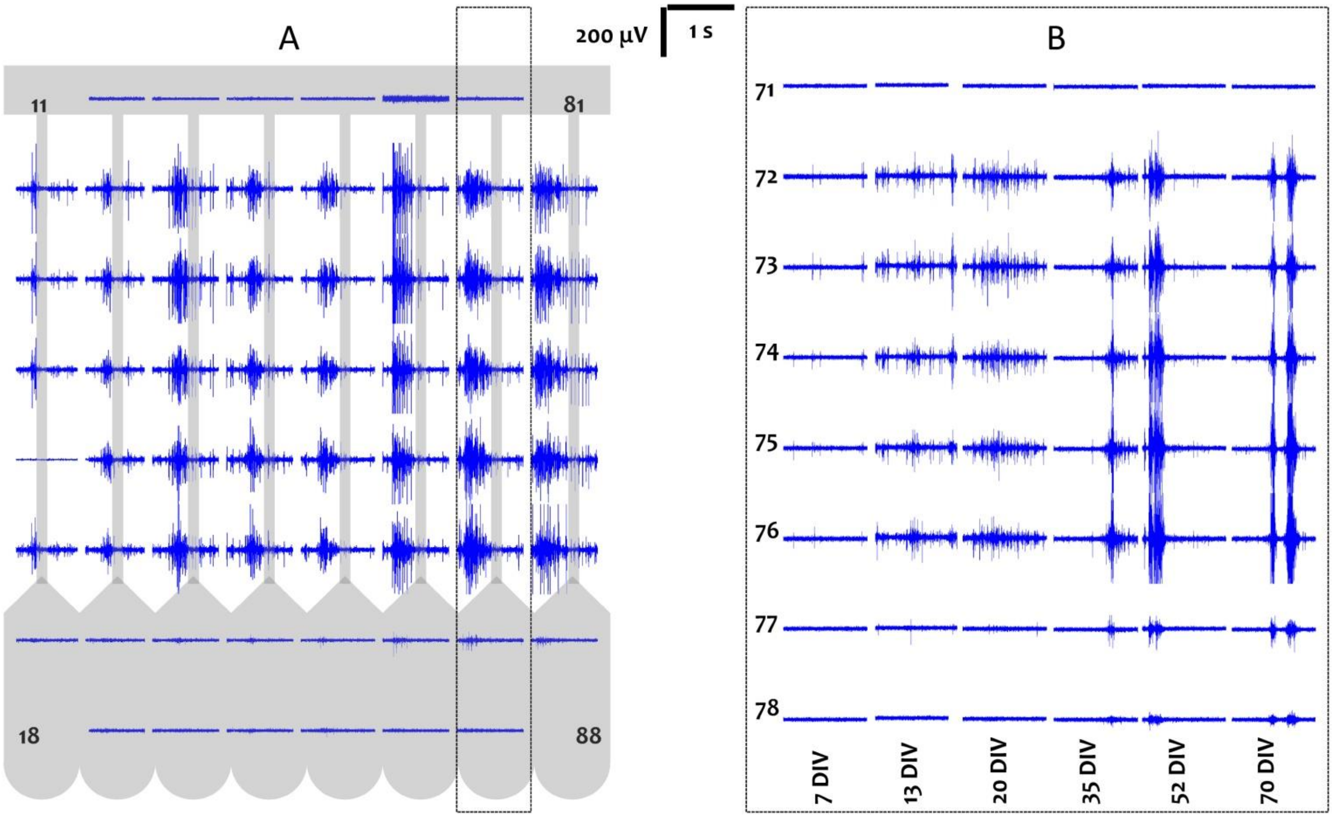


**Suppl. Fig. 3** Spatial and temporal approaches for electrophysiological activity readout. A) *Simultaneous recording from all network compartments* including 8 reservoirs and 8 microchannels at 10 DIV. One-second activity window from each electrode at the same instance. The activity in each somal module was recorded by two electrodes, except for two modules in the lower right and left corners. Five electrodes recorded activity along each microchannel at different positions. Due to the higher electrical intra-channel resistivity, axonal activity amplitudes were notably higher than those recorded from the electrodes in the reservoir modules (two bottom rows). Activity in the somal reservoir increased at later DIVs due to higher network complexity upon maturation. The last row of electrodes in the counterpart reservoir did not record any activity, which suggests that the axonal extracellular potential is too weak to be recorded by electrodes located in areas without physical confinements, although microscopy pictures confirmed that in almost all cases axons passed over these electrodes in the counterpart reservoir (Suppl. Fig. 2 and 4). B) *Long-term recording over months from one module* including a reservoir and a microchannel shows the activity evolution in a specific network area over time (dashed rectangle in A).

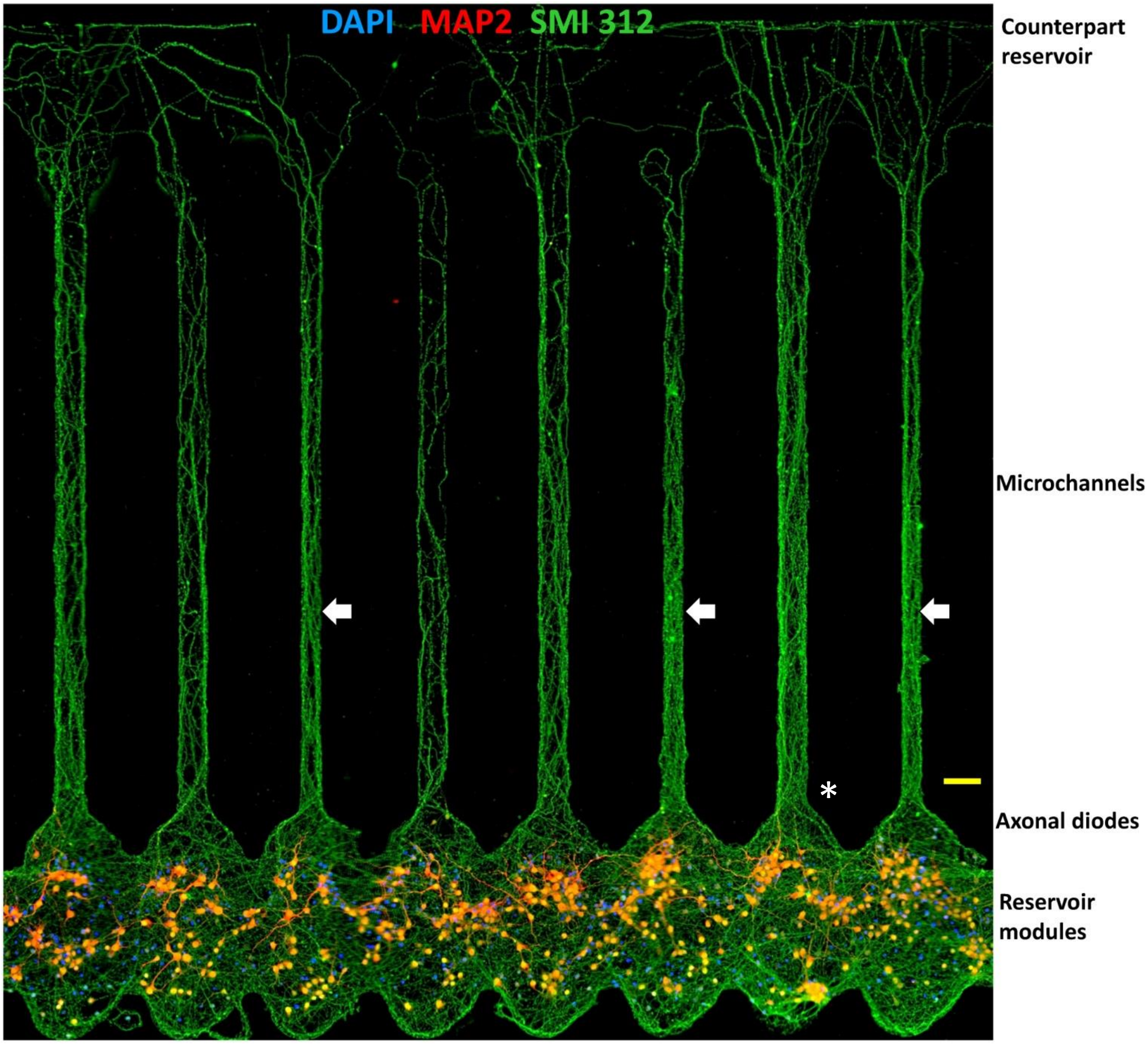


**Suppl. Fig. 4** Immunofluorescence image of a cortical culture on a coverslip at 14 DIV. The merged image shows nuclei (DAPI, blue), somata and dendrites (MAP2, red) and axons (SMI 312, green). Scale bar: 50 µm. Axonal projections growing in *thin* microchannels (25 µm) are shown by white arrows. A magnified view of the module marked by an asterisk is shown in Fig. 3 in the main text.

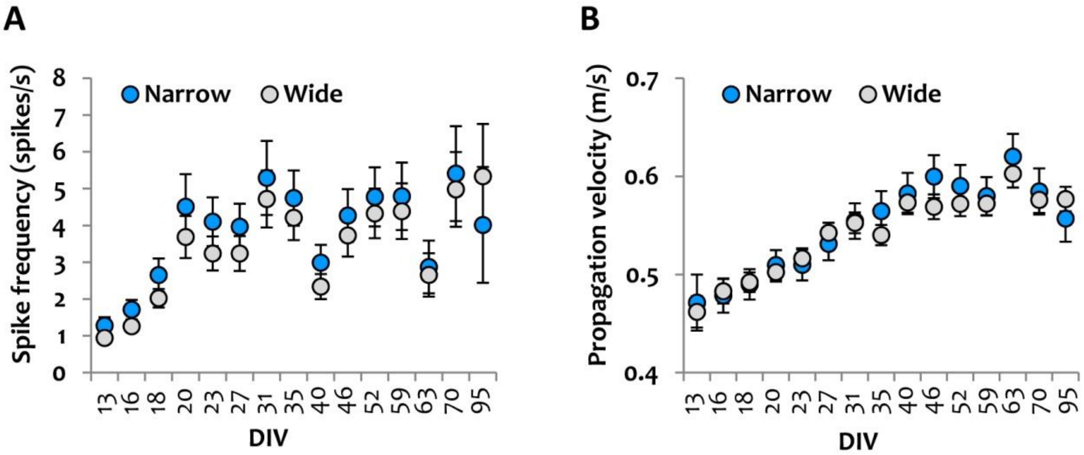


**Suppl. Fig. 5** Spike frequency and propagation velocity in narrow *vs.* wide microchannels. A) Average spike frequency in the narrow (blue circles, n= 21) and wide (gray circles, n= 35) microchannels at different DIVs. The activity in each module (narrow or wide microchannels) at each recording DIV was averaged across all MEAs and was represented as mean ± S.E.M. B) Average propagation velocity in the narrow (blue circles, n= 12) and wide (gray circles, n= 30) microchannels over the whole length of the study. The velocity for each microchannel type (narrow or wide) at each recording DIV was averaged across all MEAs (n=7) and was represented as mean ± S.E.M. For further details on the calculations, please see Suppl. Table 4.

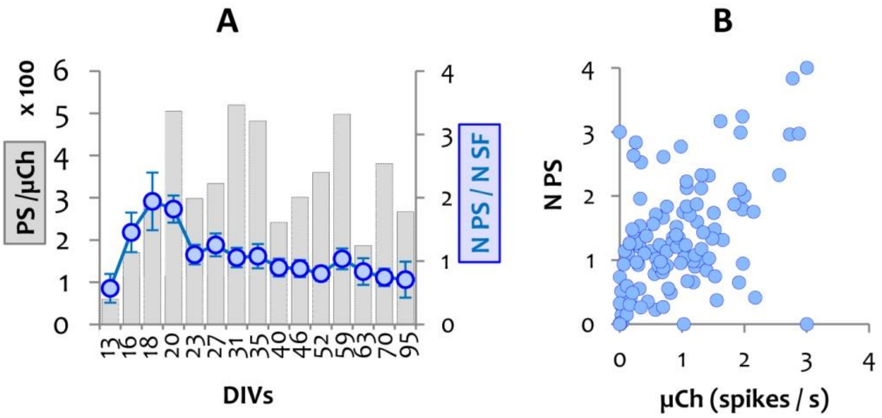


**Suppl. Fig. 6** Propagating spikes travel over the entire length of a microchannel. A) Average number of completely propagating spikes per microchannel at each day (gray bars), and normalized number of propagating spikes to spike frequency in each microchannel at different days (NPS/NSF). B) Correlation between axonal activity (spike frequency recorded from each microchannel) and normalized number of completely propagating spikes (NPS) in the same microchannel. Both the spike frequency or number of propagating spikes in each individual microchannel at each recording DIV were normalized to the average spike frequency or number of propagating spikes in the same microchannel for the whole length of the study. The correlation was performed for 840 data points including 56 microchannels at 15 different time points (recording DIV). For more details on the calculations, please see Suppl. Table 4.

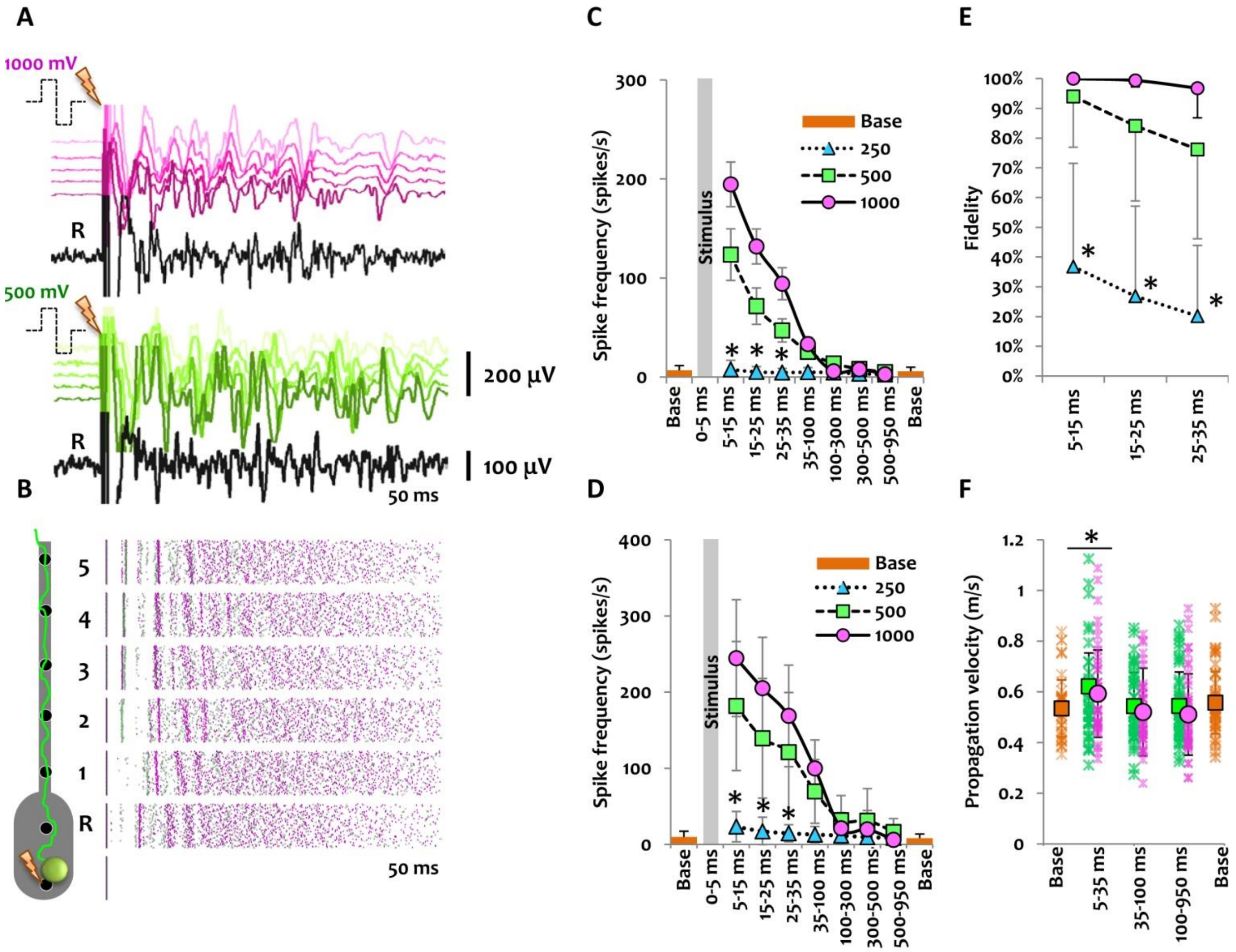


**Suppl. Fig. 7** Stimulation in the reservoir module and propagation velocity of the evoked response. A) From top to bottom: 50 ms post-stimulus responses to a symmetric biphasic (100 µs each phase, 1 Hz) stimulus with ±1,000 mV (violet), ±500mV (green) or ±250 mV (blue) amplitudes. In all cases, the stimulation pulse was applied through the bottom electrode in the reservoir module; evoked responses were recorded by the second reservoir electrode (black signal traces) and four subsequent electrodes underneath the axonal branches in the corresponding microchannels (green and red for a 500 mV and 1,000 mV stimulus, respectively). The color intensity reflects the proximity of the recording electrode to the stimulation site. B) Overlaid peri-stimulus raster plots for 100 biphasic stimulation pulses with ±1,000 mV (red) and ±500 mV (green) pulse-amplitudes over a 50 ms post-stimulation period. In each raster plot, the responses to the 100 stimuli are arranged in 100 rows from top to bottom. R: recording electrode in the reservoir. C) In the corresponding microchannel, axonal activity response to stimuli with different amplitudes in a 1 s peri-stimulus period was defined as the mean number of spikes/s in specific time-widows. The mean activity in each time-window was averaged across 12 experiments (4 cultures and 12 microchannels). In each experiment, three different stimulation amplitudes (each 180 trials; 1 Hz) were applied to the same electrode in the reservoir. Baseline activity was recorded from the same electrodes in the microchannel before and after each stimulation experiment. Data are represented as mean ± StDev. * $p < 0.05$ *vs.* baseline activity (Base) in the same microchannel. D) Activity recorded from the reservoir module (one electrode in each reservoir) for the same experiments in C. * $p < 0.05$ *vs.* baseline activity in the reservoir module. E) The response reliability to each stimulation amplitude was calculated for three peri-stimulus time windows over 180 trials together with the percentage of successful trials (at least one spike per time window). Each point represents the average reliability percentage across 12 stimulation experiments. * $p < 0.05$ *vs.* the response fidelity to 250 mV stimuli in the same time-window. F) Signal propagation velocity in axons after applying a stimulus through an electrode in the corresponding reservoir. Propagating spikes in different peri-stimulus time-windows were extracted, and then the average velocity was calculated across 12 experiments for each pulse amplitude (mean ± StDev). For each time-window, 36 points are representing the average velocity on four axonal segments between electrodes 1 to 5 in B. * $p < 0.05$ *vs.* velocity at baseline.

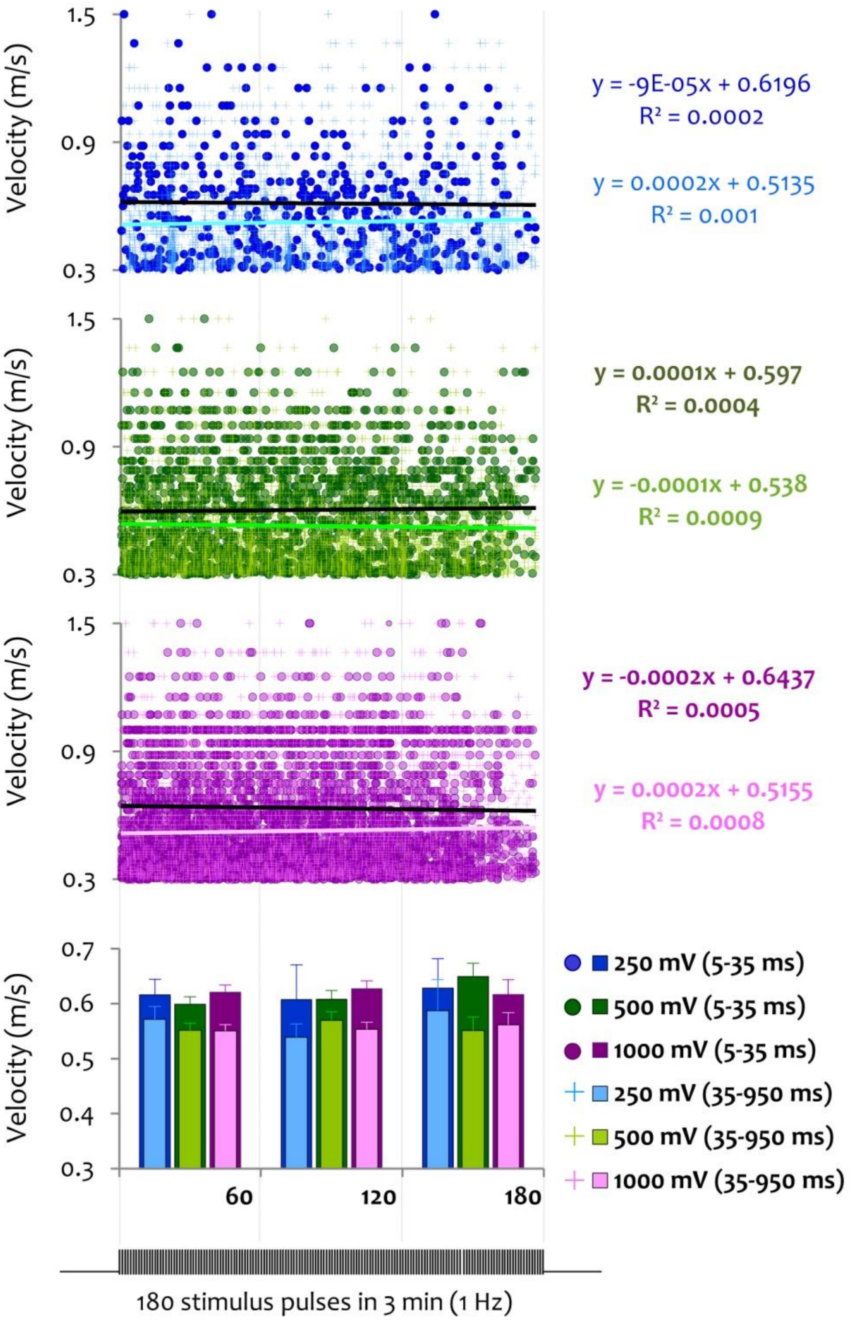


**Suppl. Fig. 8** The effect of a 1 Hz stimulus regime with 180 repetitions (3 min) on axonal conduction velocity. For each stimulus amplitude (250, 500 and 1,000 mV), the propagation velocity of a direct response (5-35 ms peri-stimulus; dark blue, dark green and dark violet disks, respectively) and a complex response (35-950 ms peri-stimulus; light blue, light green and pink crosses; respectively) are plotted above their respective stimulus time stamp. Both the scatter plots (X=stimulus time, Y=velocity of direct or evoked responses) and the correlation analysis did neither show any increase nor decrease in the signal propagation velocity as a function of the stimuli repetitions. Averaging the propagation velocity of direct or complex responses after 60, 120 and 180 stimuli repetitions showed that direct responses propagate at higher velocities when compared to complex poly-synaptic responses. The average velocity in both cases did not significantly change during 180 stimuli repetitions.

Stimulation in one reservoir module produces laterally evoked responses in the other reservoir modules. We studied the propagation of the evoked responses in the adjacent reservoir modules by averaging the evoked activity recorded from the electrodes of the other reservoirs for 5 ms windows in a 10 to 100 ms per-stimulation period (**Suppl. Fig. 9 C**). Stimulation in the left reservoir module took around 25 ms to appear in the farthest reservoir module located in the right corner at a 1,400 µm distance. In contrast, the peak of the evoked responses in the reservoirs close to the stimulation site appeared earlier, *e.g.* after 10 ms in an adjacent reservoir at 200 µm distance from the stimulation point (**Suppl. Fig. 9 C**). Considering a five milliseconds time delay at each synaptic node, there should be at least five synaptic connections between the neurons in the left-corner reservoir module and the neurons in the right-corner reservoir module. Stimulation in a central reservoir module led to lateral propagation toward left and right reservoirs with large latency in the distant reservoir modules (**Suppl. Fig. 9 C**). Regarding a 10 ms to 15 ms delay between adjacent reservoirs, it can be assumed that the neurons in neighbor subpopulations were wired through two to three synaptic connections (**Suppl. Fig. 9 C**). It was also possible to monitor the propagation properties along the axons in the microchannels, which are indirectly excited by stimulating a neuron in other reservoir modules (**Suppl. Fig. 9 A**). While stimulating in the corresponding reservoir provided a sharp stimulus-locked propagating feature at 5 ms to 10 ms peri-stimulus, stimulation in a neighbor reservoir showed complex and non-packed propagation features in the same microchannel (**Suppl. Fig. 9 B**).

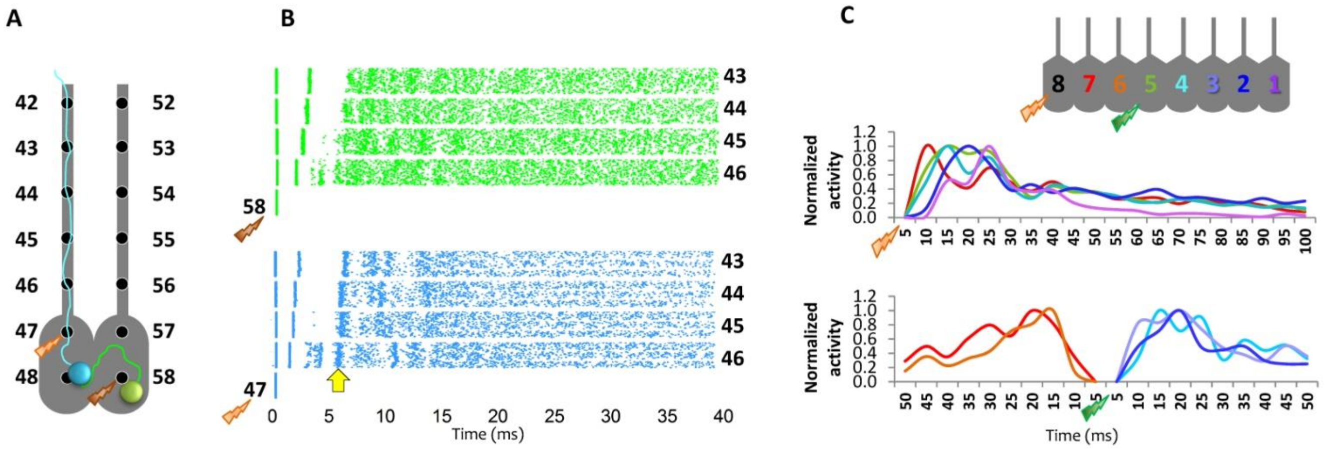


**Suppl. Fig. 9** Tracking the stimulation-evoked response in other network modules. A) Schematic view of two adjunct modules for a stimulation (500 mV, first-positive biphasic, 1 Hz) in one reservoir module and an adjacent module. The stimulated electrodes are pointed out by an orange flash. B) Peri-stimulus raster plot of recorded activity (40 ms) on four subsequent electrodes of a microchannel in response to stimulation in its corresponding reservoir module (blue; bottom) and adjunct reservoir module (green, top). In each raster plot, the responses to the 100 stimuli were arranged in 100 rows from top to bottom. The direct and dense propagating response starting around 5 ms after stimulation in the corresponding reservoir module (yellow arrow) is absent for stimulation in the adjunct reservoir. C) Evoked activity propagation in response to the stimulation in a central (green) or marginal (black) reservoir module. The spike frequency on two electrodes of each reservoir module counted in 5 ms peri-stimulus intervals up to 100 ms (upper graph, stimulation on corner) or 50 ms (lower graph, stimulation in central reservoir) and averaged for these intervals. The results from each interval were normalized to the maximum activity recorded for that interval and plotted for each reservoir over time.

**Suppl. Table 1** Medium exchange, recording and microscopy time lines in cultures that were used in long-term activity and velocity studies

| *Day in vitro* | 01 | 02 | 03 | 04 | 05 | 06 | 07 | 08 | 09 | 10 | 11 | 12 | 13 | 14 | 15 | 16 | 17 | 18 | 19 | 20 | 21 | 22 | 23 | 24 | 25 | 26 | 27 | 28 | 29 | 30 | 31 | 32 | 33 | 34 | 35 | 36 | 37 | 38 | 39 | 40 | 41 | 42 | 43 | 44 | 45 | 46 | 47 | 48 | 49 | 50 |
|---|---|---|---|---|---|---|---|---|---|---|---|---|---|---|---|---|---|---|---|---|---|---|---|---|---|---|---|---|---|---|---|---|---|---|---|---|---|---|---|---|---|---|---|---|---|---|---|---|---|---|
| Electrophysiology recording | | | | | | | | | ■ | | ■ | | ■ | | | ■ | | ■ | | | ■ | | ■ | | | | ■ | | | | ■ | | | | ■ | | | | | ■ | | | | | | ■ | | | | |
| Microscopy | | | | | | ■ | | | ■ | | ■ | | ■ | | | ■ | | ■ | | | ■ | | ■ | | | | ■ | | | | ■ | | | | ■ | | | | | ■ | | | | | | ■ | | | | |
| Medium exchange | | | | | | ■ | | | | | | | ■ | | | | | | | ■ | | | | | | | | ■ | | | | | | ■ | | | | | | | | ■ | | | | | | | ■ | |

| *Day in vitro* | 51 | 52 | 53 | 54 | 55 | 56 | 57 | 58 | 59 | 60 | 61 | 62 | 63 | 64 | 65 | 66 | 67 | 68 | 69 | 70 | 71 | 72 | 73 | 74 | 75 | 76 | 77 | 78 | 79 | 80 | 81 | 82 | 83 | 84 | 85 | 86 | 87 | 88 | 89 | 90 | 91 | 92 | 93 | 94 | 95 | 96 | 97 | 98 | 99 |
|---|---|---|---|---|---|---|---|---|---|---|---|---|---|---|---|---|---|---|---|---|---|---|---|---|---|---|---|---|---|---|---|---|---|---|---|---|---|---|---|---|---|---|---|---|---|---|---|---|---|
| Electrophysiology recording | | ■ | | | | | | | ■ | | | | ■ | | | | | | | ■ | | | | | | | | | | | | | | | | | | | | | | | | | ■ | | | | |
| Microscopy | | ■ | | | | | | | ■ | | | | ■ | | | | | | | ■ | | | | | | | | | | | | | | | | | | | | | | | | | ■ | | | | |
| Medium exchange | | | | | ■ | | | | | | | | ■ | | | | | | | ■ | | | | | | | | | | ■ | | | | | | | | ■ | | | | | | | | | | | |

Recording preceded microscopy on those days with both experiments. Medium was exchanged after recording or microscopy if necessary.

**Suppl. Table 2** Correlation results between activity features and propagation velocity

| Parameter | n | Pearson r | 95% confidence interval | P value (two-tailed) | $R^2$ |
|---|---|---|---|---|---|
| **Spike frequency (spikes/s)** | 423 | 0.05 | -0.04 to 0.14 | **0.31** | 0.00 |
| **Burst frequency (bursts/s)** | 415 | 0.11 | 0.01 to 0.20 | **0.03** | 0.01 |
| **Spikes in burst (%)** | 416 | 0.31 | 0.22 to 0.39 | **< 0.0001** | 0.10 |
| **Burst duration (s)** | 416 | 0.23 | 0.13 to 0.31 | **< 0.0001** | 0.05 |
| **Number of spikes in burst (n)** | 416 | 0.21 | 0.12 to 0.30 | **< 0.0001** | 0.05 |
| **Inter-spike interval in burst (s)** | 416 | -0.09 | -0.18 to 0.00 | **0.05** | 0.01 |
| **Spike frequency in burst (spikes/s)** | 416 | 0.28 | 0.18 to 0.36 | **< 0.0001** | 0.08 |
| **Inter-burst interval (s)** | 416 | -0.09 | -0.18 to 0.00 | **0.07** | 0.01 |

**Suppl. Table 3** DIV-based correlation analysis between activity features and velocity (only *p*-values are included)

| DIV | SF | BF | %S in B | B du | S in B (n) | ISI in B | SF in B | IBI |
|---|---|---|---|---|---|---|---|---|
| **13** | 0.624 | 0.740 | 0.485 | | 0.323 | 0.668 | | 0.855 |
| **16** | 0.702 | 0.872 | 0.580 | 0.416 | 0.746 | 0.984 | 0.373 | 0.826 |
| **18** | 0.339 | 0.802 | 0.165 | 0.020 | 0.032 | 0.108 | 0.646 | 0.336 |
| **20** | 0.035 | 0.089 | 0.082 | 0.728 | 0.739 | 0.398 | 0.289 | 0.433 |
| **23** | 0.075 | 0.065 | 0.183 | 0.128 | 0.250 | 0.372 | 0.446 | 0.116 |
| **27** | 0.237 | 0.167 | 0.359 | 0.318 | 0.474 | 0.246 | 0.445 | 0.288 |
| **31** | 0.370 | 0.931 | 0.077 | 0.253 | 0.258 | 0.187 | 0.045 | 0.953 |
| **35** | 0.230 | 0.258 | 0.109 | 0.255 | 0.274 | 0.292 | 0.089 | 0.501 |
| **40** | 0.176 | 0.151 | 0.055 | 0.048 | 0.123 | 0.145 | 0.347 | 0.014 |
| **46** | 0.126 | 0.163 | 0.072 | 0.102 | 0.090 | 0.066 | 0.060 | 0.298 |
| **52** | 0.941 | 0.846 | 0.042 | 0.216 | 0.210 | 0.301 | 0.552 | 0.940 |
| **59** | 0.208 | 0.280 | 0.041 | 0.066 | 0.069 | 0.087 | 0.060 | 0.054 |
| **63** | 0.974 | 0.881 | 0.382 | 0.473 | 0.730 | 0.394 | 0.684 | 0.888 |
| **70** | 0.814 | 0.278 | 0.453 | 0.462 | 0.118 | 0.937 | 0.685 | 0.610 |
| **95** | 0.254 | 0.144 | 0.399 | 0.829 | 0.752 | 0.248 | 0.574 | 0.127 |

SF: mean spike frequency, BF: mean burst frequency, %S in B: mean percentage of spikes per burst, B du: mean burst duration, S in B (n): mean number of spikes per burst, ISI in B: mean inter-spike intervals in burst, SF in B: mean spike frequency in burst, IBI: mean inter-burst intervals

**Suppl. Table. 4** Data treatment and averaging in different Figures

| Figure | Variable | Parameters | Formula |
|---|---|---|---|
| **Fig. 4 & Fig. 5** | Mean spike frequency at each DIV in somata or axons | ***$SF_{DIVi}$***: Mean spike frequency (spikes/s) on each individual electrode *i* of each MEA at each DIV. ***$SF_{av\_comp}$***: Activity was recorded from the electrodes in the same compartment category (µCh or somal modules) *c* and was averaged across all *n* MEAs. | $SF_{av\_comp} = \frac{1}{n \cdot \#c} \sum_{j=1, i \in c}^{n} (SF_{DIVi})$ |
| **Fig. 4 & Fig. 5** | Mean burst frequency at each DIV in somata or axons. Other burst features were treated in the same way | ***$BF_{DIVi}$***: Mean burst frequency (bursts/s) on each individual electrode *i* of each MEA at each recording DIV. ***$BF_{av\_comp}$***: Burst activity was recorded from electrodes in the same compartment category (µChor somal modules) *c* and was averaged across all *n* MEAs. | $BF_{av\_comp} = \frac{1}{n \cdot \#c} \sum_{j=1, i \in c}^{n} (BF_{DIVi})$ |
| **Suppl. Fig. 6** | Normalized spike frequency (NSF) in each µCh at each DIV | ***$SF_{DIV\mu Ch}$***: Mean spike frequency (spikes/s) on 5 electrodes in each *µCh* of each MEA at each recording DIV. ***$SF_{av\_\mu Ch}$***: Averaged ***$SF_{DIV\mu Ch}$*** for all DIVs. ***$NSF_{DIV\mu Ch}$: $SF_{DIV\mu Ch}$*** divided by ***$SF_{av\_\mu Ch}$*** | $NSF_{DIV\mu Ch} = \frac{SF_{DIV\mu Ch}}{SF_{av\_\mu Ch}}$ |
| **Suppl. Fig. 6** | Normalized number of completely propagating spikes (NPS) in each µCh at each DIV | ***$PS_{DIV\mu Ch}$***: Total number of propagating spikes, which traveled over the entire length of the µCh in each *µCh* of each MEA at each recording DIV. ***$PS_{av\_\mu Ch}$***: Averaged ***$PS_{DIV\mu Ch}$*** for all DIVs in each µCh ***$NPS_{DIV\mu Ch}$: $PS_{DIV\mu Ch}$*** divided by ***$PS_{av\_\mu Ch}$*** | $NPS_{DIV\mu Ch} = \frac{PS_{DIV\mu Ch}}{PS_{av\_\mu Ch}}$ |
| **Fig. 5** | Mean propagation velocity at each DIV | ***$PV_{DIVi}$***: Mean propagation velocity (m/s) in each µCh for all completely propagated spikes at each DIV. ***$PV_{av}$*** **:** ***$PV_{DIVi}$*** was averaged across all *n* MEAs (*m*= *n*×8 µChs) at each recording DIV. | $PV_{av} = \frac{1}{m \cdot \#c} \sum_{j=1, i \in c}^{n} (PV_{DIVi})$ |
| **Fig. 5 F** | Mean propagation velocity at each DIV in proximal or distal axons | ***$PV_{DIVi\text{-}PD}$***: Mean propagation velocity (m/s) in proximal or distal sections of each µCh for all completely propagating spikes at each DIV. ***$PV_{av\_PD}$***: ***$PV_{DIVi\text{-}PD}$*** was averaged across all *n* MEAs (*m*= *n*×8 µChs) at each recording DIV. | $PV_{av\_PD} = \frac{1}{m \cdot \#c} \sum_{j=1, i \in c}^{n} (PV_{DIVi\_PD})$ |
| **Fig. 7 E & Suppl. Fig. 7 E** | Mean fidelity percentage in response to the stimulation amplitude | ***$\%F_i$***: in each experiment (*i*), the percentage of the positive responses to a stimulus was calculated by dividing the number of positive responses by 180 (number of stimulation trials). ***$\%F_{av}$***: Average fidelity percentage calculated by averaging %C over *n* experiments | $\%F_{av} = \frac{1}{n} \sum_{j=1, i \in c}^{n} (\%Fi)$ |

µCh: Microchannel

To understand if spikes in bursts that were detected on subsequent electrodes originated from the same source, we initially resorted to different spike sorting protocols (*e.g.* principal component analysis, spike area matching, spike slope comparison).However, none of these allowed us to separate individual units satisfactorily, mainly because most of the axons in a bundle were located in almost the same position with respect to the electrode and because extracellularly recorded spike shapes from the very same neuron may considerably vary as a result of various geometrical and electrode-related factors. Checking the spike train data visually confirmed that currently available spike sorting methods are not powerful enough to reliably and automatically identify and separate individual units. We therefore wrote a MATLAB (MathWorks) script that implemented a simple detection strategy based on the following requirements. Propagating spikes had to travel over the *entire microchannel length* and had to stay within a *time delay of 0 ms to 2 ms* between each 200 µm-pitched electrode pair. We checked the functionality of the method both manually (visually) and also by shuffling the data between subsequent electrodes. Shuffling was performed temporally (by introducing random time shifts for one or more electrodes), spatially (by changing the electrode arrangement) and by combining these two shuffling strategies. The maximum rate for falsely detecting random spikes as propagating spikes from shuffled datasets rarely exceeded 10%. This result confirmed with acceptable confidence that the script-extracted propagating spikes in the original data were not randomly detected signals from different sources. Although this detection strategy reduced the number of positive hits and thus the scope of the dataset, it eliminated any ambiguity by not only excluding all spikes, which failed to propagate completely, but also those with missing time stamps on at least one electrode. The latter may be related to geometrical changes in axonal morphology (*e.g.* because the axon (bundle) did not cross the electrode sufficiently closely).

**M File 1** Find all propagating spike timestamps on 5 subsequent electrodes and export them as 5 parallel columns for separate microchannels (Excel sheets).

```
address = 'C:\ ……..';
th =0.002;  % Defines the maximum range of the time delay
dirlist = dir(fullfile(address,'*Ex*.xlsx')); % Exports the selected timestamps into the Excel file
num_files = length(dirlist);

clear finalData

for numtext =1:num_files  % Source Excel file name
  disp(['processing excel file--------' num2str(dirlist(numtext).name) '----'])
  [~,sheets] = xlsfinfo(fullfile(address,dirlist(numtext).name));
  clear finalData;

  for shnum = 1:length(sheets)    % Excel sheet name
    chnum = sheets{shnum};       % Microchannel (ch) name
    disp(['processing sheet file--------' chnum '----'])
    num1 = xlsread(fullfile(address,dirlist(numtext).name),chnum);

    generalData= num1(2:end,1:4);
    [c,r] = find(isnan(generalData));
    generalData(c,r)=-1;

    %-----------------------------------------------------------

    clear finalData1;

    data1=[];
    for numlevel =1:size(generalData,1)
      tmp=zeros(1,size(generalData,2));
      idx = numlevel;
```

```
            tmp(1,1) = generalData(numlevel,1);
            for i = 1:size(generalData,2)-1
                if(~isempty(idx))
                    a1 = generalData(idx(1),i)> generalData(:,i+1);
                    b1 = (generalData(idx(1),i)-th) <= generalData(:,i+1);
                    idx = find(a1& b1);

                    if(~isempty(idx))
                        tmp(1,i+1) = generalData(idx(1),i+1);
                    end

                end

            end
            data1 = [data1;tmp];

        end
        [m,n]= find(data1==0);
        data1(unique(m),:)=[];

 xlswrite(fullfile(address,dirlist(numtext).name), data1, chnum, 'G2')

    end
end
```

**M File 2** With respect to the timestamp of each stimulation pulse, the script finds the timestamps of the stimulus-induced propagating spikes (0-950 ms peri-stimulus) on five/four subsequent electrodes and exports them as 5 parallel columns for separate microchannels (Excel sheets).

```
address = 'C:\.......';
dirlist = dir(fullfile(address,'S*.xlsx'));
num_files = length(dirlist);
clear finalData
for numtext =1:num_files     % Source Excel file name
    disp(['processing excel file--------' num2str(dirlist(numtext).name) '----'])
    [~,sheets] = xlsfinfo(fullfile(address,dirlist(numtext).name));
    clear finalData;
    for shnum = 1:length(sheets)       % Excel sheet name
        chnum = sheets{shnum};          % Microchannel (ch) name
        disp(['processing sheet file--------' chnum '----'])
        num1 = xlsread(fullfile(address,dirlist(numtext).name),chnum);

        levelData = num1(:,7:13);
        generalData= num1(:,1:5);
        levelData(isnan(levelData))=0;
        %----------------------------------------------------------
        clear finalData1;
        for i = 1:size(levelData,2)-1
            i
            data1=[];
            for numlevel =1:size(levelData,1)
                a = generalData(:,5) > levelData(numlevel,i);
                b =generalData(:,5) <= levelData(numlevel,i+1);
                idx = find((a.*b)==1);
                if(~isempty(idx))
                    for jj =1:length(idx)
                        data1= [data1;generalData(idx(jj),:)];
                    end
                end
            end

if(~isempty(data1))
            switch i
                case 1
                    xlswrite(fullfile(address,dirlist(numtext).name), data1, chnum, 'o2')
```

```
            case 2
              xlswrite(fullfile(address,dirlist(numtext).name), data1, chnum, 'u2')
            case 3
              xlswrite(fullfile(address,dirlist(numtext).name), data1, chnum, 'aa2')
            case 4
              xlswrite(fullfile(address,dirlist(numtext).name), data1, chnum, 'ag2')
            case 5
              xlswrite(fullfile(address,dirlist(numtext).name), data1, chnum, 'am2')
            case 6
              xlswrite(fullfile(address,dirlist(numtext).name), data1, chnum, 'as2')
          end

end
data_time_delay = Timedelay_write(data1,i,'ba2','bf2','bk2','bp2','bu2','bz2',[address,'\',dirlist(numtext).name],chnum);
data_velocity =
Velocity_write(data_time_delay,i,'cg2','cl2','cq2','cv2','da2','df2',[address,'\',dirlist(numtext).name],chnum);
          finalData1{i} = data1;
        end
        finalData{numtext,shnum} =finalData1;
    end
end

xlswrite(fullfile(address,dirlist(numtext).name), data1, chnum, 'o2')
```

## Videos

Movie 1 · 3D animation of a microchannel device on a MEA with a structured neural network (https://youtu.be/YlDgU47n6cY)

Movie 2 · Time lapse video (17 h) of network modules (https://youtu.be/HMBNHep4OuY)

Movie 3 · Snapshots of network morphology at different DIVs (https://youtu.be/eySuy-vSP0A)

Movie 4 · Collection of 10 s signal profiles at different DIVs from one individual module (reservoir and microchannel) (https://youtu.be/hyZX1yk_q6o)

Movie 5 · Overlapped signals recorded from subsequent electrodes and their time-delay between electrodes (https://youtu.be/UaShA7pROo4)
